# An Interdisciplinary Examination of Stress and Injury Occurrence in Athletes

**DOI:** 10.3389/fspor.2020.595619

**Published:** 2020-12-14

**Authors:** Harry Fisher, Marianne JR Gittoes, Lynne Evans, C Leah Bitchell, Richard J Mullen, Marco Scutari

**Affiliations:** ^1^Cardiff School of Sport and Health Sciences, Cardiff Metropolitan University, Cardiff, United Kingdom; ^2^Division of Sport, Health and Exercise Sciences, Department of Life Sciences, Brunel University, Uxbridge, United Kingdom; ^3^Istituto Dalle Molle di Studi sull'Intelligenza Artificiale (IDSIA), Lugano, Switzerland

**Keywords:** sports injury, stress, interdisciplinary, Bayesian network, sports psychology

## Abstract

This paper adopts a novel, interdisciplinary approach to explore the relationship between stress-related psychosocial factors, physiological markers and occurrence of injury in athletes using a repeated measures prospective design. At four data collection time-points, across 1-year of a total 2-year data collection period, athletes completed measures of major life events, the reinforcement sensitivity theory personality questionnaire, muscle stiffness, heart rate variability and postural stability, and reported any injuries they had sustained since the last data collection. Two Bayesian networks were used to examine the relationships between variables and model the changes between data collection points in the study. Findings revealed muscle stiffness to have the strongest relationship with injury occurrence, with high levels of stiffness increasing the probability of sustaining an injury. Negative life events did not increase the probability of injury occurrence at any single time-point; however, when examining changes between time points, increases in negative life events did increase the probability of injury. In addition, the combination of increases in negative life events and muscle stiffness resulted in the greatest probability of sustaining an injury. Findings demonstrated the importance of both an interdisciplinary approach and a repeated measures design to furthering our understanding of the relationship between stress-related markers and injury occurrence.

## Introduction

Over the last four decades sport-related injuries have received increased research attention (Ivarsson et al., 2017) in response to the high incidence (Rosa et al., 2014; Sheu et al., 2016) and associated undesirable physical and psychological effects (Leddy et al., 1994; Brewer, 2012). Multiple psychological (Slimani et al., 2018), anatomical (Murphy et al., 2003), biomechanical (Neely, 1998; Hughes, 2014), and environmental (Meeuwisse et al., 2007) factors have been associated with sports injury occurrence and several models of injury causation have been proposed that highlight the multifactorial nature of injury occurrence (Meeuwisse et al., 2007; Kumar, 2001; Wiese-Bjornstal, 2009), of which one of the most widely cited was developed by Andersen and Williams (1988) and Williams and Andersen (1998).

Williams and Andersen's (1998) stress-injury model proposed that when faced with a potentially stressful athletic situation, an athlete's personality traits (e.g., hardiness, locus of control, and competitive trait anxiety), history of stressors (e.g., major life events and previous injuries) and coping resources (e.g., general coping behaviours) contribute to the injury response, either interactively or in isolation. The stress response is central to the model and reflects the bi-directional relationship between athletes' appraisal of, and response to, a stressful athletic situation. The model predicts that athletes who have a history of stressors, personality traits that intensify the stress response and few coping resources, will exhibit greater attentional (e.g., peripheral narrowing) and/or physiological (e.g., increased muscle tension) responses that put these individuals at greater injury risk (Supplementary Figure 1).

Within Williams and Andersen's (1998) model, major life events, which is a component of an athlete's history of stressors, most consistently predicts injury occurrence (Williams and Andersen, 2007); specifically, major life events with a negative, as opposed to positive, valence (Passer and Seese, 1983; Maddison and Prapavessis, 2005). Personality traits and coping resources have also been found to predict injury with athletes more likely to sustain an injury if they have poor social support and psychological coping skills, and high trait anxiety and elevated competitive state anxiety; compared to those with the opposing profile (Smith et al., 1990; Lavallée and Flint, 1996; Ivarsson and Johnson, 2010). However, the amount of variance explained by these psychosocial factors has been modest and typically between 5 and 30% (Ivarsson and Johnson, 2010; Galambos et al., 2005), which indicates a likely interaction with other factors.

While the psychosocial factors in Williams and Andersen's (1998) model have received the most research attention, less insight into the mechanisms through which these factors are proposed to exert their effect exists. To elaborate, the model suggests that injuries are likely to occur through either increased physiological arousal resulting in increased muscle tension and reduced flexibility or attentional deficits caused by increased distractibility and peripheral narrowing. However, to date, the research has largely focused on attentional deficits (Andersen and Williams, 1999; Rogers and Landers, 2005; Wilkerson, 2012; Swanik et al., 2007). For example, Andersen and Williams (1999) found athletes with high life event stress coupled with low social support had greater peripheral narrowing under stressful conditions compared to athletes with the opposing profile; these athletes went on to sustain an increased number of injuries during the following athletic season. Rogers and Landers (2005) further supported Andersen and Williams's (1999) earlier findings by identifying that peripheral narrowing under stress mediated 8.1% of the relationship between negative life events and injury.

Knowledge of the physiological factors (e.g., increased muscle tension and reduced motor control) contributing to the remaining variance between negative life events and athletic injury remains sparse (Williams and Andersen, 1998). One challenge faced by researchers addressing the sports injury problem through a psychological lens is the multifactorial nature of injury, and the possible interaction with physiological factors in the stress response (Meeuwisse et al., 2007; Wiese-Bjornstal, 2009). For example, a large body of research has suggested that training-related stress is also likely to contribute to the stress response and injury occurrence (Lee et al., 2017; Djaoui et al., 2017) and may account for the unexplained variance from the psychological predictors. In an attempt to combine the psychosocial factors proposed by Williams and Andersen (1998) and potential markers of training-related stress, Appaneal and Perna (2014) proposed the biopsychosocial model of stress, athletic injury and health (BMSAIH) to serve as an extension to Williams and Andersen's (1998) model. The BMSAIH enhances our understanding of the mediating pathways between the stress response and injury alongside other health outcomes and behavioural factors that impact sports participation (Appaneal and Perna, 2014). The central tenet of the BMSAIH is that psychosocial distress (e.g., negative life events) may act synergistically with training-related stress as a result of high-intensity and high-volume sports training, and “widen the window of susceptibility” (Appaneal and Perna, 2014) to a range of undesirable health outcomes including illness and injury. Consequently, the BMSAIH provides an important framework that has enhanced insight into the multi-faceted nature of the injury process by building on Williams and Andersen's (1998) model whilst including other physiological markers of training-related stress.

The BMSAIH offers researchers several potential avenues for research. To date, the physiological mechanisms explored using the BMSAIH have focused on the hormonal response to high intensity training and injury. For example, Perna and McDowell (1995) examined life event stress and cortisol response in athletes following an exhaustive graded exercise test. Participants were split into high and low life event stress groups, and the high life event stress group were found to have both higher cortisol in response to the graded exercise test, and increased symptomatology, including muscle complaints and viral illness, over the 30 days following the graded exercise test; however, Perna and McDowell (1995) did not explicitly examine the relationship between cortisol response to high intensity training and sports injury. Research from a wider physiological perspective has identified other important training-related stress markers that have been associated with an increased risk of injury. Postural stability, skeletal muscle characteristics and heart rate variability are of particular interest here as they have been examined in a variety of different stress-related disciplines including psychopathology, lifestyle, and geriatric research (Ockenburg et al., 2015; Gervasi et al., 2017; Bailey et al., 2013; Rath and Rath, 2017). Importantly, these variables have also been linked to injury occurrence in athletes (Pickering-Rodriguez et al., 2017; Plews et al., 2012; Trojian and McKeag, 2006; Williams et al., 2017) but have typically been studied in isolation without an assessment of the interaction with the psychosocial factors that are known to contribute to injury. Furthermore, a reliance on designs that capture a single point of measurement precludes the assessment of intra- as well as inter-individual changes and the effect of the time interval between measurement and injury occurrence on subsequent injuries (Johnson et al., 2014). Such an approach fails to capture changes in both psychosocial factors and physiological markers that may occur preceding an injury. Importantly, a repeated measurement approach would enable an assessment of how variables and their interactions change with respect to time and would provide greater insight into the effect that repeated exposure to major life events and other stress-related factors has on injury occurrence.

Recently, Bittencourt et al. (2016) advocated a move away from studying isolated risk factors and instead, adopt a complex systems approach in order to understand injury occurrence. Such an approach posits that injury may arise from a complex “web of determinants” (Bittencourt et al., 2016), where different factors interact in unpredictable and unplanned ways, but result in a global outcome pattern of either adaptation or injury. Capturing the uncertainty and complexity of the relationships between different variables using an appropriate interdisciplinary analysis within the framework of a complex systems approach is challenging. Bayesian network (BN) modelling provides one solution by allowing the construction of graphical probabilistic models using the underlying structure that connects different variables (Scutari and Denis, 2014). The learned BN structure can be used for inference by obtaining the posterior probabilities of a particular variable for a given query (e.g., if the value of variable A is x and the value of variable B is y, what is the probability variable C of being value z?). Furthermore, unlike regression or structural equation models, BN's do not distinguish between dependent and independent variables when the underlying relationship in the network may not be known (Olmedilla et al., 2018). BN modelling subsequently provides a valuable but underused interdisciplinary approach to investigating the complex and unpredictable interactions of psychosocial and physiological factors implicated in the injury process.

Using the frameworks provided by Williams and Andersen's (1998) stress injury model and Appaneal's BMSAIH model (Appaneal and Perna, 2014), the aim of this longitudinal interdisciplinary study was to develop new understanding of the multifaceted interactions of psychosocial (life event stress and reinforcement sensitivity) and physiological stress-related factors (heart rate variability, muscle stiffness, and postural control) with injury occurrence. Due to the inevitable constraints of a longitudinal interdisciplinary study of this kind, we have not been able to incorporate a number of other variables that might also be of interest (e.g., coping resources, emotional states, and attentional deficits). A prospective, repeated measures design incorporating the physiological and psychosocial measures combined with BN modelling was used to address the study aim.

## Materials and Methods

### Ethics Statement

Ethical approval was obtained from the University ethics committee prior to the start of the study and all participants provided informed consent.

### Participants

A total of 351 athletes (male: *n* = 231, female: *n* = 120) were initially recruited for the study from a British University and local sports clubs (Table 1). Participants had an average age of 22.0 ± 7.0 years and represented a range of team (football, rugby, netball, cricket, lacrosse, basketball, and field hockey) and individual sports (running, tennis, weightlifting, gymnastics, judo, swimming, and golf). Participants' self-rated competitive level ranged from recreational to international standard. They were engaged in training for their respective sports for at least 5 h per week. A total of 162 (46%) participants had sustained an injury in the 12 months prior to the start of the study (male: *n* = 114 [49%], female; *n* = 48 [40%]). All participants were injury free (no modifications to their usual training routine due to a sport related medical problem for a minimum of 4 weeks) at the start of the study.

**Table 1:** Participant characteristics.

	**Female (*n* = 120)**	**Male (*n* = 231)**
**Demographics M (SD)**		
Age (yrs)	26.0 (11.3)	20.2 (1.8)
Height (cm)	167.4 (7.6)	177.8 (7.8)
Body mass (kg)	67.0 (9.5)	82.0 (14.6)
Training hours per week	8.5 (4.5)	11.2 (8.8)
**Current competitive level** ***n*** **(%)**		
Recreational	3 (4)	7 (4)
University	45 (56)	141 (80)
National/International	33 (41)	28 (16)

### Study Design

The study adopted a prospective, repeated measures design that included a total of four data collection sessions over a 12-month period. The first three data collections were conducted in person, with the final collection (injury reporting) being performed remotely via email (Supplementary Figure 3). The primary dependant variable was injury outcome (injured or non-injured), with major life events, the reinforcement sensitivity theory personality questionnaire, muscle stiffness, balance ability and heart rate variability serving as independent variables. The following sections describe the specific measures used and outline the procedure for both the data collection and analysis.

### Measures

#### Injury

At each data collection, participants self-reported all injuries they sustained during the study period via a questionnaire. An injury was defined as any sports related medical problem causing the athlete to miss or modify their usual training routine for at least 24 h (Fuller et al., 2006, 2007; Timpka et al., 2014). Minor scrapes and bruises that may require certain modifications (e.g., strapping or taping) but did not limit continued participation were not considered injuries (Appaneal et al., 2009). Injury status (did/did not sustain an injury) served as the main outcome measure.

#### Major Life Events

A modified version of the Life Events Survey for Collegiate Athletes (LESCA) was used to measure participants' history of life event stress (Petrie, 1992). The LESCA is the most widely used measure of major life events for athletes in the sports injury literature. Modifications were made to the LESCA to ensure the suitability of the items for the study cohort (Supplementary Table 1). The LESCA comprises 69 items that reflect possible life events that participants may have experienced. Example items include, “Major change in the frequency (increased or decreased) of social activities due to participation in sport,” “Major change in the amount (more or less) of academic activity (homework, class time, etc.),” and “Major change in level of athletic performance in actual competition (better or worse).” Participants were asked to rate the perceived impact of each life event they had experienced within 12-months preceding the study onset on an 8-point Likert scale anchored at −4 (*extremely negative*) and +4 (*extremely positive*). Negative life event (NLE) and positive life event (PLE) scores were calculated by summing the negative and positive scores, respectively. A score for total life events (TLE) was also calculated by summing the absolute values for both negative and positive events. Petrie (1992) reported test-retest reliabilities at 1-week and 8-weeks with values ranging from 0.76 to 0.84 (*p* < 0.001) and 0.48 to 0.72 (*p* < 0.001), respectively. Petrie also provided evidence of discriminant, convergent, and predictive validity. For this study, composite reliability (Fornell and Larcker, 1981) was preferred to Cronbach's alpha as it does not assume parallelity (i.e., all factor loadings are constrained to be equal, and all error variances are constrained to be equal) and instead takes into consideration the varying factor loadings of the items in the questionnaire. The composite reliability for the LESCA in this study was 0.84.

#### Reinforcement Sensitivity Theory Personality Questionnaire

A revised version of the Reinforcement Sensitivity Theory Personality Questionnaire (RST-PQ) was used to measure motivation, emotion, personality and their relevance to psychopathology (Corr and Cooper, 2016). The revised version of the RST-PQ comprises 51 statements that measure three major systems: Fight-Flight-Freeze System (FFFS; e.g., “I am the sort of person who easily freezes-up when scared”), Behavioural Inhibition System (BIS; e.g., “When trying to make a decision, I find myself constantly chewing it over”) and four Behavioural Approach System (BAS) factors; Reward Interest (e.g., “I regularly try new activities just to see if I enjoy them”), Goal Drive Persistence (e.g., “I am very persistent in achieving my goals”), Reward Reactivity (e.g., “I get a special thrill when I am praised for something I've done well”), and Impulsivity (e.g., “I find myself doing things on the spur of the moment”). Participants rated each item on a scale from 1 (*not at all*) to 4 (*highly*) to reflect how well each statement described their personality in general. The responses to items associated with each subscale (FFFS, BIS, RI, GDP, RR, and I) were summed to give a total personality score that was subsequently used for further analysis. The composite reliabilities for each subscale were; BIS = 0.92, FFFS = 0.77, GDP = 0.87, I = 0.71, RI = 0.77, RR = 0.81. Further details regarding the revised RST-PQ are in Supplementary Appendix 1.

#### Heart Rate Variability

A Polar V800 heart rate monitor (HRM) and Polar H7 Bluetooth chest strap (Polar OY, Finland) was used to collect inter-beat interval (IBI) data. IBI recordings using the Polar V800 are highly comparable (ICC = 1.00) with ECG recordings (Giles et al., 2016), which are considered the gold standard for assessing heart rate variability (HRV). In addition, HRV indices calculated from IBI and ECG data have shown a strong correlation (*r* = 0.99) in athletes (Caminal et al., 2018) and under spontaneous breathing conditions (Plews et al., 2017).

#### Musculoskeletal Properties

A handheld myometer (MyotonPRO, Myoton AS, Tallinn, Estonia) was used to measure passive muscle stiffness. The MyotonPRO is a non-invasive, handheld device that applies a mechanical impulse of 0.40 N for 0.15 ms perpendicular to the surface of the skin. The impulse causes natural damped oscillations in the tissue, which are recorded by a three-axis digital accelerometer sensor in the device. The raw oscillation signal is then processed, and the stiffness parameter is calculated (Agyapong-Badu et al., 2016). The MyotonPRO has previously been reported to be a reliable and valid tool for the measurement of *in-vivo* tissue stiffness properties (Chuang et al., 2013; Pruyn et al., 2016; Nair et al., 2014), and has demonstrated good internal consistency (coefficient of variation < 1.4%) over sets of 10 repetitions (Aird et al., 2012).

#### Postural Stability

Postural stability was assessed with a modified version of the balance error scoring system (mBESS) based on the protocol recommended by Hunt et al. (2009). In total, each trial of the mBESS was performed without shoes (McCrory et al., 2013) and included six stances in the following order; dominant leg (DL; standing on the dominant foot with the non-dominant foot at approximately 30-degrees of hip flexion and 45-degrees of knee flexion), non-dominant leg (NDL; standing on the non-dominant foot with the dominant foot at approximately 30-degrees of hip flexion and 45-degrees of knee flexion) and tandem leg stance (TS; standing heel-to-toe with the non-dominant foot behind the dominant) on firm and foam (Alcan airex AG, Sins, Switzerland) surfaces, respectively (Supplementary Figure 2). To determine leg dominance, participants were asked their preferred leg to kick a ball to a target, and the chosen limb was labelled as dominant (van Cingel et al., 2017). Participants were asked to maintain each stance for a total of 20-s. Participants hands were placed on hips at the level of the iliac crests. A Sony DSC-RX10 video camera (Sony Europe Limited, Surrey, United Kingdom) was used to record each participants' performance during the mBESS.

The error identification criteria from the original BESS protocol was used by the lead researcher who scored all the BESS trials. One error was recorded if any of the following movements were observed during each trial: (a) lifting hands off iliac crests; (b) opening eyes; (c) stepping, stumbling, or falling; (d) moving the thigh into more than 30 degrees of flexion or abduction; (e) lifting the forefoot or heel; and (f) remaining out of the testing position for more than 5-s (Riemann et al., 1999). A maximum score of 10 errors was possible for each stance. Multiple errors occurring simultaneously were recorded as one error. A participant was given the maximum score of 10 if they remained out of the stance position for more than 5-s. A total score was calculated by summing the total number of errors recorded on all stances (DL, NLD, and TS, on foam and firm surfaces). To assess the intra-rater reliability, a single measurement, absolute agreement, two-way mixed effects model for the intraclass correlation (Koo and Li, 2016) was used on a sample of 40 participants from the first time point. The test-retest scoring of BESS resulted in a “good” to “excellent” ICC score (ICC = 0.93, 95% confidence interval = 0.88–0.96), indicating the scoring was reliable.

### Procedure

At the start of the academic year (September), coaches of sports teams at a British University and local sports clubs were contacted and informed about the study. With the coaches' permission, the lead researcher attended training sessions to inform athletes about the overall purpose of the study and the requirements of participation. Athletes who met the participation criteria and volunteered to take part in the study were invited to attend scheduled testing sessions. A repeated measures prospective cohort design was used to assess athletes' major life events, stress-related physiological markers and injury status over a 2-year period. Within the study period, each participant was asked to complete a total of four data collections, with each data collection separated by a 4-month interval (Supplementary Figure 3). Participants provided informed consent before data collection commenced.

For each of the data collections (T1, T2, and T3), participants followed the same protocol in a specific order (Supplementary Figure 4). To ensure all measures could be collected within a viable time-frame, participants were separated into two groups. The first group completed all computer-based measures followed by all physical measurements, whereas the second group completed all physical measurements followed by computer-based measures. Participants were randomly assigned to one of the two groups and remained in those groups across all time points.

#### Questionnaires

The questionnaires, which included demographic information, the LESCA, RST-PQ (T1, T2, T3) and injury status (T2, T3, T4) were completed on-line (SurveyMonkey Inc., USA, www.surveymonkey.com). The instructions for the LESCA were modified at T2 and T3 so that participants reported major life events that had occurred since the previous testing session. For injury reporting, participants were asked to record any injuries that they had sustained since the last data collection. The data were downloaded from surveymonkey.com and imported into R (R Core Team, 2019) for analysis.

#### Heart Rate Variability

To minimise potential distractions, participants were directed to a designated quiet area in the laboratory where IBI data were recorded. Participants were instructed to turn off their mobile devices to avoid any interference with the Bluetooth sensor. Each chest strap was dampened with water and adjusted so it fitted tightly but comfortably, as outlined by Polar's guidelines. Participants were seated and asked to remain as still as possible for the duration of the recording. No attempt was made to control participants' respiratory frequency or tidal volume (Denver et al., 2007). Inter-beat interval (IBI) data was collected for 10-min at a sampling frequency of 1,000 Hz.

Raw, unfiltered IBI recordings were exported from the Polar Flow web service as a space delimited .txt file and imported into R (R Core Team, 2019) where the *RHRV* package (Rodriguez-Linares et al., 2019) was used to calculate HRV indices. Raw IBI data was filtered using an adaptive threshold filter, and the first 3-min and last 2-min of each recording were discarded, leaving a 5-min window that was used to calculate the root mean square of successive differences (RMSSD) in RR intervals following the recommendations for short term IBI recordings (Laborde et al., 2017; Malik et al., 1996). RMSSD was calculated as:

(1) $$\begin{array}{r} \bar{RR}=\frac{1}{N}\overset{n}{\underset{i=1}{\sum }}RR_{i} \end{array}$$

Where N is the length of the time series, and *RR*_*i*_ the RR interval between beats *i* and *i* − 1, where each beat position corresponds to the beat detection instant.

#### Muscle Stiffness

To assess muscle stiffness, participants lay horizontally on a massage bed and four testing sites were identified on each lower limb. The muscle belly of the rectus femoris (RF), biceps femoris (BF), medial gastrocnemius (MG), and lateral gastrocnemius (LG) sites were identified using a visual-palpatory technique to determine the exact location of each site (Chuang et al., 2012). The visual-palpatory technique required the participant to contract the target muscle to aid the lead researcher to visually identify the muscle. The participant was then asked to relax the muscle and the muscle was palpated to locate the muscle belly. A skin safe pen (Viscot all skin marker pen, Viscot Medical LLC, NJ) was used to mark the testing site in the center of the muscle belly.

After the eight testing sites had been identified, the testing end of the MyotonPRO (diameter = 3 mm) was positioned perpendicular to the skin on the testing site. A constant pre-load of 0.18 N was applied for initial compression of subcutaneous tissues. The device was programmed to deliver five consecutive impulses, separated by a 1-s interval (Morgan et al., 2018). For each impulse, the device computed passive stiffness values, with the median of the five values being saved by the device for further analysis. In accordance with Myoton.com, a set of five measurements with a coefficient of variation (CV) of <3% was accepted. Sets of measurements above 3% were measured again to ensure data reliability. Measurements were uploaded using MyotonPRO software and imported in R (R Core Team, 2019) for further analysis. For each participant, the sum of all eight testing sites was calculated to provide a total lower extremity stiffness score and was used for further analysis.

#### Postural Stability

Instructions for the mBESS were read to each participant and a demonstration of the positions was provided by the research assistant. For each position, participants were instructed to close their eyes, rest their hands on their iliac crests and remain as still as possible for 20-s. Participants were instructed to return to the testing position as quickly as possible if they lost their balance. The video recording was started prior to the first stance position and stopped after all stances had been completed. Each completed mBESS protocol took approximately 4-min. Only one trial was performed to avoid familiarisation effects across the repeated measurement (Valovich et al., 2003). The video recordings for each participant were imported from the recording equipment (Sony DSC-RX10) and the lead researcher scored each trial using the error identification criteria.

### Data Analysis

Two Bayesian Networks (BN) were used to explore the relationships between the psychosocial measures, physiological markers of stress, and sports injury. A BN is a graphical representation of a joint probability distribution among a set of random variables, and provides a statistical model describing the dependencies and conditional independences from empirical data in a visually appealing way (Scutari and Denis, 2014). A BN consists of arcs and nodes that together are formally known as a directed acyclic graph (DAG), where a node is termed a parent of a child if there is an arc directed from the former to the latter (Pearl, 1988). However, the direction of the arc does not necessarily imply causation, and the relationship between variables are often described as probabilistic instead of causal (Scutari and Denis, 2014). The information within a node can be either continuous or discrete, and a complete network can contain both continuous and discrete nodes; however, discrete networks are the most commonly used form of BN (Chen and Pollino, 2012). In discrete networks, conditional probabilities for each child node are allocated for each combination of the possible states in their parent nodes and can be used to assess the strength of a dependency in the network.

In order to use discrete networks, continuous variables must first be split into categorical levels. When there are a large number of variables in the network, limiting the number of levels has the benefit of producing a network that is more parsimonious in terms of parameters. For example, a network with 10 variables each with two levels has 100 (10^2) possible parameter combinations, however the same network with three levels has 1,000 (10^3) possible parameter combinations, the latter being significantly more computationally expensive. Using a larger number of splits in the data also comes at a cost of reducing the statistical power in detecting probabilistic associations, and reduces the precision of parameter estimates for the probabilistic associations that are detected because it reduces the sample-size-to-parameters ratio (Scutari and Denis, 2014). Typically, no more than three levels have been used in Bayesian networks in the sports injury literature (Olmedilla et al., 2018; Ruiz-Pérez et al., 2019)

Learning the structure of the network is an important step in BN modelling. The structure of a network can be constructed using expert knowledge and/or data-driven algorithm techniques (e.g., search and score, such as hill climbing and gradient descent algorithms; Scutari and Denis, 2014). The learned structure can then be used for inference by querying the network^1^ and obtaining the posterior probabilities of a particular node for a given query. The posterior distribution can be obtained by *Pr*(*X*|*E, B*)=*Pr*(*X*|*E, G*, Θ), where the learned network *B* with structure *G* and parameters Θ, are investigated with new evidence *E* using the information in *B* (Scutari and Denis, 2014). When a network contains many nodes, the outcome of a particular node can be assessed conditional on the states of any subset of nodes in the network. BNs therefore provide a unique and versatile approach to modelling a set of variables to uncover dependency structures within the data.

BNs have recently been used in the sport psychology literature (Fuster-Parra et al., 2017; Olmedilla et al., 2018; Ruiz-Pérez et al., 2019) and offer several benefits over traditional statistical analysis. For example, predictions can be made about any variable in the network, rather than there being a distinction between dependent and independent variables in the data, such as in linear regression models that are often used within the sport psychology literature (Bittencourt et al., 2016; Olmedilla et al., 2018). Furthermore, the structure of a network can be obtained from both empirical data *and* prior knowledge about the area of study; the latter being particularly useful when there are a large number of variables in the network, or only a small number of observations are available in the data (Xiao-Xuan et al., 2007). In such instances, a purely data driven approach to learning the network would be time-consuming due to the large parameter space, and inefficiency at identifying an approximation of the true network structure. Prior knowledge about dependencies between variables can therefore be included in the network structure, while still allowing a data driven approach for unknown dependencies, to improve the overall computation of the network structure (Heckerman et al., 1995; Xu et al., 2015). The following sections detail the steps taken in the current study to firstly prepare the data for each network, and then obtain the structure of each network that was used for further inference.

#### First Network

##### Data preparation

Of the 351 participants that were initially recruited for the study, 94 only completed the first time point, and therefore had to be removed from the study as no injury information was obtained for these participants following the first time point. To prepare the data for the BN, missing values in the dataset were first imputed. Out of the 650 total measurements across all time points in the current study, there were 31 (4.77%) missing muscle stiffness measurements and 70 (10.77%) missing heart rate recordings. The missing data were due to technical faults in the data collection equipment and were considered to be missing completely at random. A missing rate of 15–20% has been reported to be common in psychological studies, and several techniques are available to handle missing values (Enders, 2003; Lang et al., 2013). In the current study, the *caret* R package (Kuhn et al., 2008) was used to impute the missing values. A bagged tree model using all of the non-missing data was first generated and then used to predict each missing value in the dataset. The bagged tree method is a reliable and accurate method for imputing missing values in data and is superior to other commonly used methods such a median imputation (Kuhn et al., 2008).

A median split technique was used to discretise the data used in the network into “Low” and “High” levels. All variables apart from negative and total life events were approximately normally distributed and required no further transformation prior to the median split. For the LESCA questionnaire data, a cumulative total of the current, and previous time points was calculated at each time point to account for the potential continuing effect of the life events experienced by athletes over time. Given the limited support for a relationship between positive life events and injury (Williams and Andersen, 2007), only negative and total life events were included in the network. Cumulative negative, and cumulative total life event scores at each time point were first log scaled so distributions were approximately normal, and then binarised using the median at each time point. In addition to the log scaled cumulative values, an untransformed negative life event score from the first time point (baseline NLE) was included as an additional variable based on previous literature that indicates this variable should have a strong relationship with injury outcome (Ivarsson et al., 2017).

##### Network structure

To obtain the network structure, several steps were taken to ensure that both a theoretically realistic network, and a network that was an appropriate fit to the collected data, was used for inference. Prior knowledge about the network structure was included by providing a list of arcs that are always *restricted* from being in the network (blacklist), and a list of arcs that are always *included* in the network (whitelist). Additionally, there are several scoring functions such as Bayesian Information Criteria (BIC) and Bayesian Dirichlet equivalent uniform (BDeu) that can be used to compare network structures with certain nodes and arcs included or excluded (Scutari and Denis, 2014). To account for the repeated measures design employed in this study and to maximise the use of the data, pairs of complete cases (e.g., participants who completed T1 + T2, and T2 + T3) were used in a two-time Bayesian network (2TBN) structure (Murphy, 2002). In the 2TBN, variables measured at T2 could depend on variables measured at T1 (e.g., T1 → T2) and variables measured at T3 could depend on variables measured at T2 (e.g., T2 → T3). However, arcs were blacklisted between T2 → T1 and T3 → T2 to preserve the order in which data was collected. Variables were separated into two groups; “explanatory,” for variables that were fixed (e.g., gender), or “independent,” for variables that were measured at each time point and could vary during the study. Independent variable names were suffixed with _1 for time point T, and _2 for time point T+1 (e.g., T1_1 → T2_2 and T2_1 → T3_2). Formatting the data in this way meant participants who completed T1 and T2, but did not complete T3, could still be included in the analysis. In addition to the blacklisted arcs between T2 → T1 and T3 → T2, the direction of arcs was restricted between independent variables and explanatory variables (e.g., independent → explanatory); however, arcs were not restricted between explanatory → independent variables. Finally, arc direction was restricted between specific nodes within the explanatory variables. Arcs from competitive level → gender, baseline NLE → gender and baseline NLE → sport type (individual or team) were included in the blacklist, as arcs in these directions did not make logical sense. All subsequent models used the same blacklist.

##### Preliminary network structures

Prior to the final network structure presented in the results section, several structures with different combinations of variables were explored. Networks were learned using a Tabu search algorithm (Russell and Norvig, 2009) and BIC was used to compare different models. A higher BIC value indicates the structure of a DAG is a better fit to the observed data (Scutari and Denis, 2014). BIC values for each combination of variables of interest are reported as the combination of variables with the highest BIC value, followed by the relative scores of the other variables in the model.

Initially, both negative life events (NLE) and total life events (TLE) were included in the network structure, however, the network score was improved when only NLE or TLE was included (NLE = −4,242.76, TLE only = −4,326.39, TLE and NLE = −4,459.23). Additionally, despite strong evidence in the literature that both NLE and TLE stress are related to injury occurrence (Williams and Andersen, 2007), network structures learned using the Tabu search algorithm failed to identify a relationship between NLE and injury or TLE and injury in the data. Given that NLE provided the highest network score, and there is a stronger relationship between negative life events and injury in the literature, an arc was whitelisted between NLE_1 and injured_1 and NLE_2 and injured_2 in the final network structure. TLE was not included in the final structure.

The subscales representing the Behavioural Activation System (Reward Reactivity [RR], Reward Interest [RI], Goal Drive Persistence [GDP] and Impulsivity [I]) showed limited connection to other variables in the network. Therefore, competing models were examined and BIC scores compared to establish the model with the best fit to the data (values are shown relative to the highest value). RI provided the highest BIC value (−3,563.13), compared to RR (−3,579.10), GDP (−3,582.39), and I (−3,582.89). Including all the variables (RR, RI, GDP, and I) resulted in a significantly lower score (−4,463.25) indicating that including all the variables was not beneficial to the model structure and did not offset the cost of the additional parameters. Therefore, only RI was included in the final structure. Finally, both total score and asymmetry (percentage difference in score between limbs) for balance were included in the initial network. However, visual inspection of the network revealed no arcs between the balance asymmetry node and any other node in the network. Therefore, balance asymmetry was removed from the final network structure. To summarise, Table 2 reports the variables that were included in the final network structure.

**Table 2:** Variables included in the final Bayesian network structure.

**Variable**	**Definition**	**State 1**	**State 2**
Competitive level	Current competitive level	Club_university_county	National_international
Gender	Gender of the participant	Female	Male
Training hours	Number of hours spent training per week	0–9 (Low)	>9–35 (High)
Sport type	Participate in an individual or team based sport	Individual	Team
Previous injury	Whether an injury had been sustained in the previous 12 months prior to the study	No Injury	Injury
Baseline NLE	Untransformed NLE at the first time point	0–13 (Low)	>13–93 (High)
FFFS	Fight-Flight-Freeze System	8–16 (Low)	>16–30 (High)
BIS	Behavioural Inhibition System	17–38 (Low)	>38–68 (High)
RI	Reward Interest	4-10 (Low)	>10–16 (High)
Stiffness	Sum of all stiffness locations	1,543–2,330 (Low)	>2,330–4,518 (High)
HRV	Root mean squared difference of successive RR intervals	2.03–4.01 (Low)	>4.01–5.94 (High)
Balance	Total balance score	5–15 (Low)	>15–46 (High)
NLE_1	Log Negative life events (NLE) at time 1	0–2.64 (Low)	>2.64–4.54 (High)
NLE_2	Log NLE at time 2	0–3.04 (Low)	>3.04–5.19 (High)
NLE_3	Log NLE at time 3	0–3.18 (Low)	>3.18–4.79 (High)
TLE_1	Log Total life events (TLE) at time 1	1.79–3.4 (Low)	>3.4–4.88 (High)
TLE_2	Log TLE at time 2	1.79–3.74 (Low)	>3.74–5.42 (High)
TLE_3	Log TLE at time 3	1.79–3.81 (Low)	>3.81–5.18 (High)

Preliminary network structures also revealed strong dependencies between the same variables at sequential time points. For example, the probability that stiffness_1 and stiffness_2 were both “High,” or both “Low” was approximately 80%. Including the arcs between the same variables from X_1 → X_2 did not provide any theoretically meaningful information to the network structure as the majority of participants would either be in a “Low” or “High” state for each pair of variables in the network. Therefore, these arcs were blacklisted from the network. To obtain the final network, the appropriate blacklist and whitelists were provided and a Tabu search algorithm identified the remaining structure of the network. The final network structure was obtained by averaging 1,000 bootstrapped models (Efron and Tibshirani, 1994) to reduce the impact of locally optimal, but globally suboptimal network learning, and to obtain a more robust model (Olmedilla et al., 2018). Arcs that were present in at least 30% of the models were included in the averaged model. The strength of each arc was determined by the percentage of models that the arc was included in, independent of the arc's direction. An arc strength of 1 indicated that the arc was always present in the network, with the value decreasing as arcs were found in fewer networks. In the respective study arcs above 0.5 were considered “significant” with arcs below 0.5 and above 0.3 “non-significant” (Scutari and Nagarajan, 2013). Arcs below 0.3 were not included in the model. The full table of arc strengths for the first and second network are available in Supplementary Tables 2, 3, respectively.

##### Network inference

Conditional probability queries (CPQ) were used to perform inference on both network structures. To conduct a CPQ, the joint probability distribution of the nodes was modified to include a new piece of evidence. The query allows the odds of a particular node state (e.g., injured_1 = “injured”) to be calculated based on the new evidence. CPQ were performed using a likelihood weighting approach; a form of importance sampling where random observations are generated from the probability distribution in such a way that all observations match the evidence given in the query. The algorithm then re-weights each observation based on the evidence when computing the conditional probability for the query (Scutari and Denis, 2014). Inference was first performed on arcs that had a strength >0.5 between the explanatory variables and independent variables and between different independent variables in the network. Of particular interest were the variables that were connected to “injured” nodes, which were examined in the network using the Markov blanket of “injured_1” and “injured_2.” A Markov blanket contains all the nodes that make the node of interest conditionally independent from the rest of the network (Fuster-Parra et al., 2017). CPQ were used to determine what effect the variables in the Markov blanket of injured nodes had on the probability of the injured node being in the “injured” state.

#### Second Network

##### Data preparation

For the second network, change scores for continuous variables between T1 → T2 and T2 → T3 were standardised to allow relative changes between variables to be compared. The “injured” variable was also modified to represent whether a participant had sustained an injury at any point over the duration of the study or were healthy for the duration of the study. Setting the data up in this way enabled the construction of a network that explicitly modelled the *amount* of change within variables between time points, as opposed to the first network that only captured changes when the median threshold was crossed from “Low” to “High.”

##### Network structure

Similar to the first network, blacklists were used to prevent arcs from independent variables → explanatory variables. In addition, baseline negative life events was dropped from the list of explanatory variables to allow the *changes* in negative life events to be the only life event variable in the network. The final network was obtained using the same approach as the first network.

##### Network inference

Conditional probability queries (CPQ) were again used to perform inference on the network structures. The Markov blanket of the “injured” node was of particular interest, and the probability of injury was investigated with combinations of variables in the Markov blanket below the mean change, at the mean change and above the mean change. Initial visual inspection of the network structure also revealed arcs between Behavioural Inhibition System (BIS) → Fight-Flight-Freeze System (FFFS) and heart rate variability (HRV) → FFFS. To investigate this relationship further, random samples were generated for BIS, HRV, and FFFS based on the conditional distribution of the nodes included as evidence in the query. The samples were then examined using a Bayesian linear regression models with the *brms* R package (Bürkner, 2017) to determine the relationship between these nodes. Weakly regularizing priors (normal prior with mean of 0 and standard deviation of 5) were used for all parameters in the model.

## Results

During the study, 46% (*n* = 117) of participants reported at least one injury with an average severity of 11 ± 31, days (range = 2–365 days). Both male and female participants reported a greater number of acute compared to chronic injuries (male, acute = 85 [69%], chronic = 39 [31%]; female, acute = 38 [72%] chronic = 15 [28%]), and non-contact injuries were more common than contact injuries (male, non-contact = 83 [67%], contact = 39 [31%]; female, non-contact = 35 [66%] contact = 18 [34%]). Table 3 shows the number and percentage of injury types sustained by both male and female participants. An additional breakdown of injury by sport, type (acute or chronic) and injury location is available in Supplementary Tables 4, 5.

**Table 3:** The number and percentage (%) of types of injuries sustained by male and female participants.

	**Female**		**Male**	
	**Lower body**	**Upper body**	**Lower body**	**Upper body**
Joint/ligament	14 (36)	5 (36)	37 (43)	14 (38)
Muscle/tendon	17 (44)	6 (43)	45 (52)	12 (32)
Other (bone, brain, and skin)	8 (21)	3 (21)	5 (6)	11 (30)

### First Network Structure

**Figure 1:**
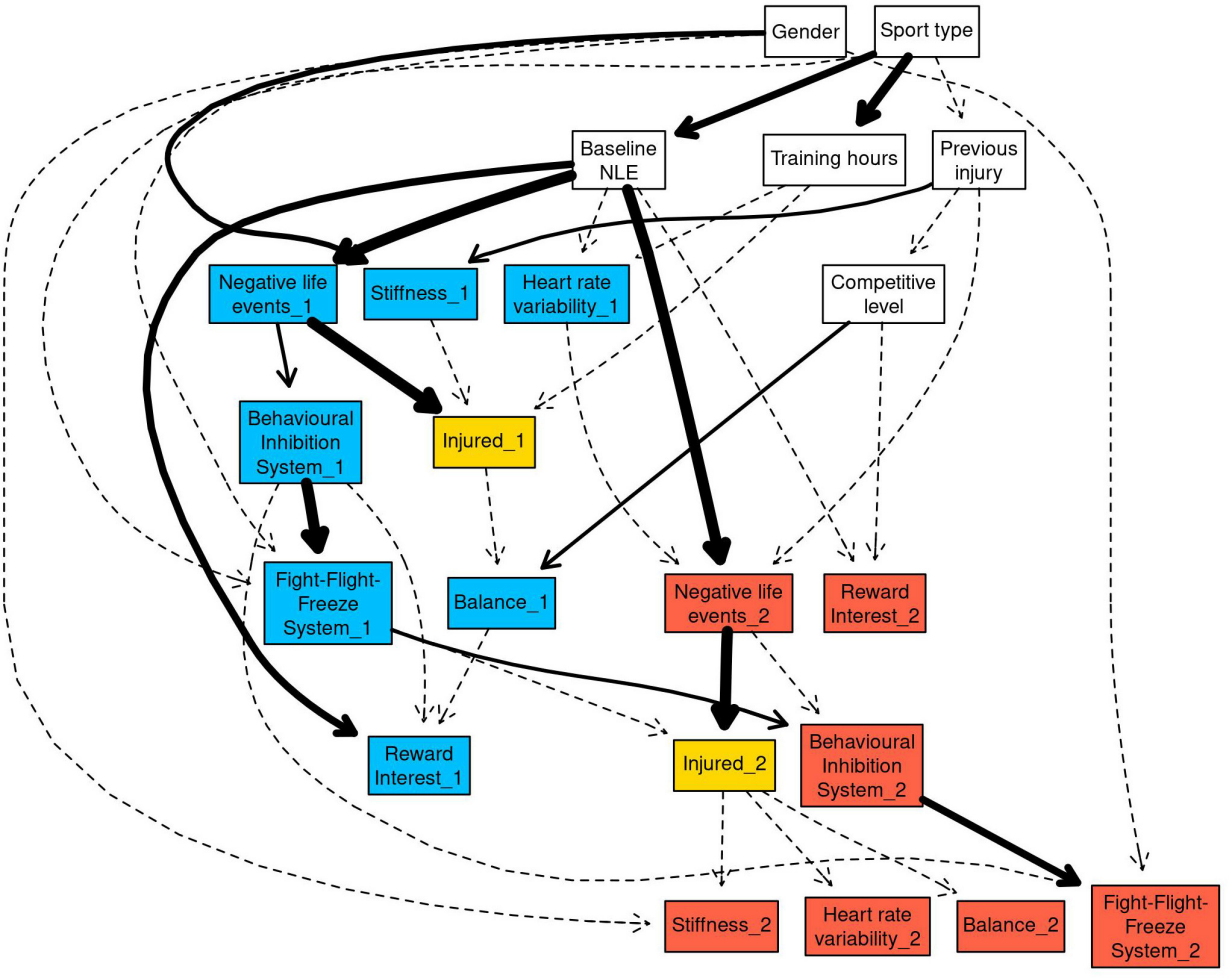
The full Bayesian network structure was plotted using the “strength.plot” function in bnlearn. The strength of each arc is shown graphically by the style of the arc. Thin, dashed arcs indicate the weakest arcs (arc strength below 0.50), whereas thick solid arcs indicate the strongest arcs (arc strength of 1). White nodes in the network indicate the explanatory variables, blue nodes indicate T1_1 and T2_1 variables, and red nodes indicated T2_2 and T3_2 variables. The injured_X nodes have been coloured gold as they are the main nodes of interest within the network.

The first network structure obtained from the data (Figure 1) examined the interactions between explanatory variables, independent variables and probability of injury across time points in the study. Strong sport-related and gender-based connections between several explanatory and independent variables were demonstrated for individual and team-based sports. The “Sport type” node had strong arcs to training hours (0.94) and baseline negative life events (NLE) (0.78). Individual athletes were more likely to have “High” training hours (0.84) compared to team-based athletes (0.60). Individual athletes were also more likely to have “High” negative life events in the 12 months preceding the start of the study compared to team-based athletes (individual athletes = 0.65, team-based athletes = 0.41). The arc from competitive level → balance_1 had a strength of 0.47, with lower level performers more likely to have decreased balance ability (0.48), compared to national level athletes (0.29). High gender-based connections were reported for the arcs from gender → stiffness_1 (0.71) and gender → stiffness_2 (0.43), with males more likely to have “High” stiffness compared to females (males = 0.62, females = 0.43). Irrespective of sport or gender, strong connections were found between explanatory variables. The arc from baseline NLE → Reward Interest (RI_1) had a strength of 0.84, and the probability of RI_1 being in the “High” state increased from 0.47 to 0.77 when baseline NLE increased from “Low” to “High.”

The first network demonstrated further strong variable interactions between high stiffness, poor balance, and injury probability. The arc from previous injury → stiffness_1 was 0.57 with athletes who reported an injury in the preceding 12 months being more likely to have “High” (0.65) compared to “Low” (0.35). stiffness. Strong arcs were present between Behavioural Inhibition System (BIS) → Fight-Flight-Freeze System (FFFS; BIS_1 → FFFS_1 = 0.98, BIS_2 → FFFS_2 = 0.74). In both instances, “High” FFFS was more likely when BIS was “High” (0.64 for _1, 0.61 for _2) compared to “Low” (0.33 for _1, 0.37 for _2). The arc between NLE → BIS had a strength of 0.55 for NLE_1 → BIS_1, and 0.37 NLE_2 → BIS_2. “Low” negative life events increased the probability of BIS being in the “High” state from 0.33 to 0.55 for NLE_1 → BIS_1, and 0.38 to 0.58 for NLE_2 → BIS_2.

#### Markov Blanket for Injured_1

**Figure 2:**
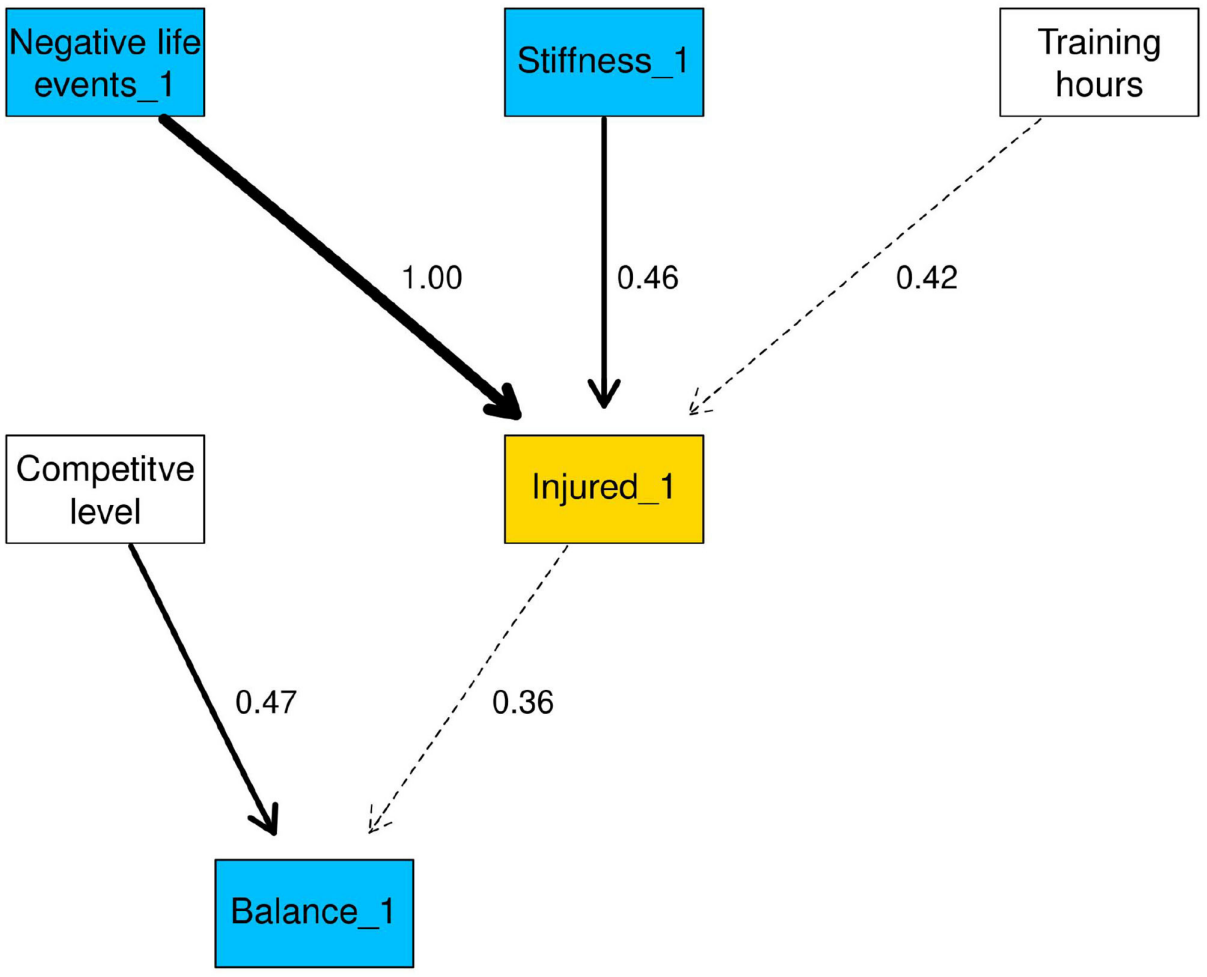
Markov blanket of injured_1. Arc strengths are included as arc labels.

The first conditional probability query (CPQ) investigated the variables in the Markov blanket for injured_1 (Figure 2), which contained hours spent training per week, negative life events (NLE_1), muscle stiffness (stiffness_1), competitive level and balance (balance_1). The arc between NLE_1 and injured_1 was fixed in the network, so has the maximum strength of 1.

The CPQ for injured_1 in the “injured” state for all variables that were linked to injured_1 is shown in Table 4. The probability of injured_1 = “injured” rose from 0.17 to 0.31 when stiffness was “High” compared to “Low”. Negative life events had a negligible effect when moving from the “Low” to “High” state.

**Table 4:** Probability of injured_1 being in the “injured” state, conditional on each variable.

**Variable**	**Low**	**High**
Balance_1	0.21	0.30
Training hours	0.18	0.28
Negative life events_1	0.24	0.26
Stiffness_1	0.17	0.31

The second CPQ investigated the outcome of injured_1 being “injured” conditional on all variables in the Markov blanket. The Markov blanket contained five nodes, each with two possible states resulting in 2^5^ combinations of variables, therefore only the three lowest and highest probabilities are shown in Table 5 (complete results in Supplementary Table 4). The combination of lower competitive level, “High” hours per week, “Low” negative life events, “High” balance and “High” stiffness resulted in a probability of 0.53 for injured_1 being in the “injured” state. When all variables were in the “Low” state the probability of “injured” was approximately 0.04.

**Table 5:** Highest and lowest probability of injured_1 being in the “injured” state, conditional on all the variables in the Markov blanket for injured_1.

**Probability**	**Competitive level**	**Training hours**	**Negative life events_1**	**Stiffness_1**	**Balance_1**
**Highest**					
0.53	club_university_county	High	Low	High	High
0.46	national_international	High	Low	High	Low
0.44	national_international	High	Low	High	High
**Lowest**					
0.06	national_international	Low	Low	Low	Low
0.05	national_international	Low	Low	Low	High
0.04	club_university_county	Low	Low	Low	Low

Negative life event stress had a negligible effect on the probability of injury, only influencing injured_1 when all other variables were fixed to “Low.” In this instance, the probability of injured_1 being “injured” rose marginally from 0.04 to 0.19, when negative life events was in the “Low” and “High” states, respectively.

#### Markov Blanket of Injured_2

**Figure 3:**
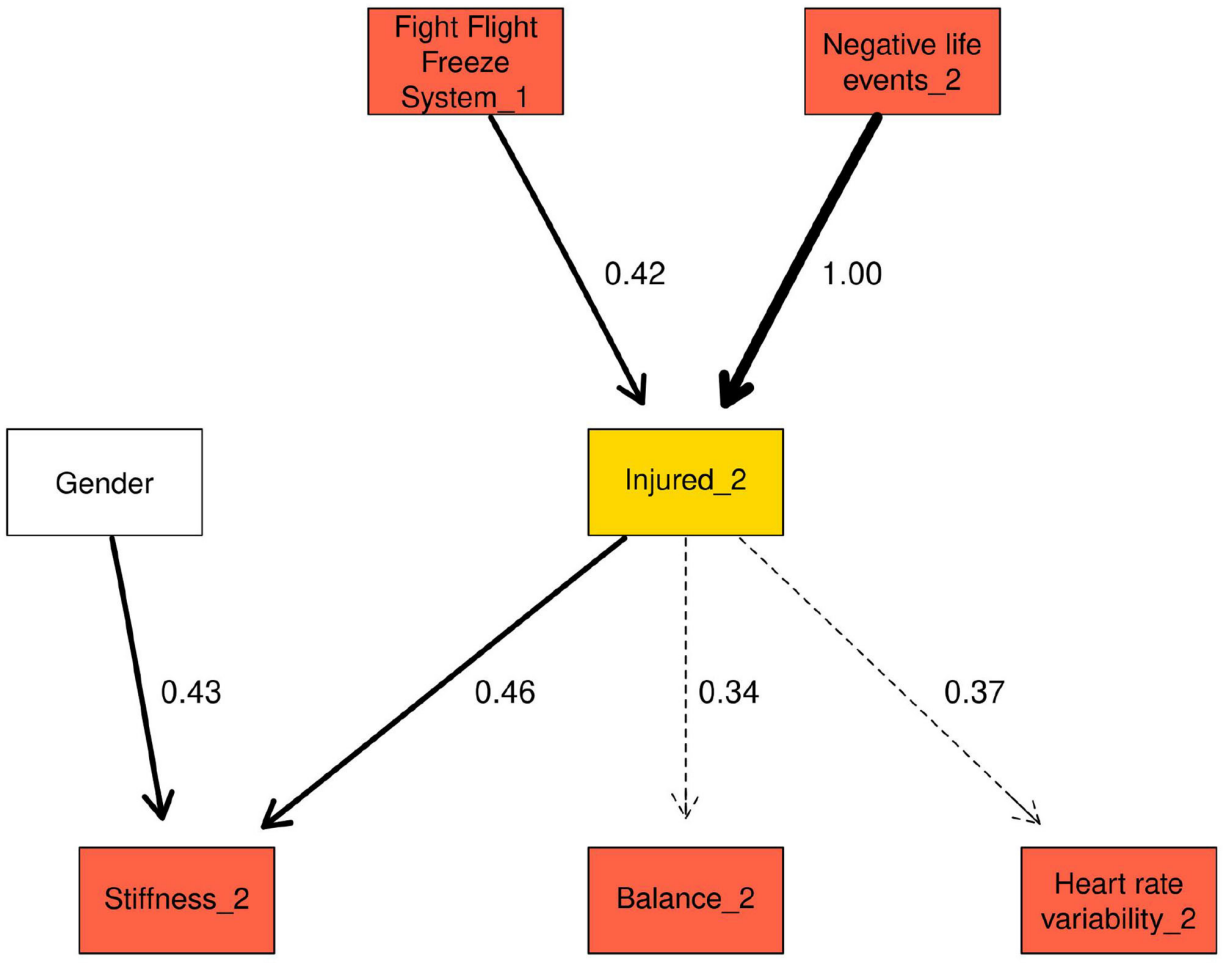
Markov blanket for injured_2.

The Markov blanket for injured_2 is shown in Figure 3 and contained gender, Fight-Flight-Freeze System (FFFS_1), stiffness_2, balance_2, and heart rate variability (HRV_2; Table 6). The arc between stiffness_2 → injured_2 was comparable to the arc between stiffness_1 → injured_1. Very weak arcs (0.3) between injured_2 → balance_2 and injured_2 → HRV_2 were also present in the Markov blanket for injured_2. Similar to injured_1, stiffness_2 doubled the probability of injured_2 being “injured” from 0.13 in the “Low” state to 0.27 in the “High” state. FFFS_1 in the “Low” state increased probability of injured_2 being “injured” by 0.19 compared to the “High” state. “High” negative life events decreased the probability of injury from 0.26 to 0.17.

**Table 6:** Probability of injured_2 being in the “injured” state, conditional on each variable in the Markov blanket for injured_2.

**Variable**	**Low**	**High**
Balance_2	0.17	0.27
Fight-Flight-Freeze System_1	0.30	0.11
Heart rate variability_2	0.26	0.17
Negative life events_2	0.23	0.19
Stiffness_2	0.13	0.27

The three lowest and highest conditional probabilities based on all the variables in injured_2 Markov blanket are presented in Table 7 (complete results in Supplementary Table 5). The combination of “Low” FFFS_1, “High” stiffness_2, “High” balance resulted in the greatest probability of injured_2 being “injured,” with the highest probability of injury being 0.53. With all other variables held in the “High” state, the probability of injured_2 being “injured” rose from 0.15 to 0.35 when FFFS_1 was in the “Low” compared to “High” state. The combination of “Low” stiffness, “Low” balance and “High” FFFS resulted in the lowest probability of injured_2 being “injured”.

**Table 7:** Highest and lowest probability of injured_2 being in the “injured” state, conditional on all the variables in the Markov blanket for injured_2.

**Probability**	**Fight-Flight-Freeze System_1**	**Negative life events_2**	**Stiffness_2**	**Heart rate variability_2**	**Balance_2**
**Highest**					
0.53	Low	Low	High	Low	High
0.46	Low	High	High	Low	High
0.41	Low	Low	High	High	High
**Lowest**					
0.06	High	High	Low	Low	Low
0.05	High	Low	Low	High	Low
0.04	High	High	Low	High	Low

### Second Network Structure—Changes Within Variables

**Figure 4:**
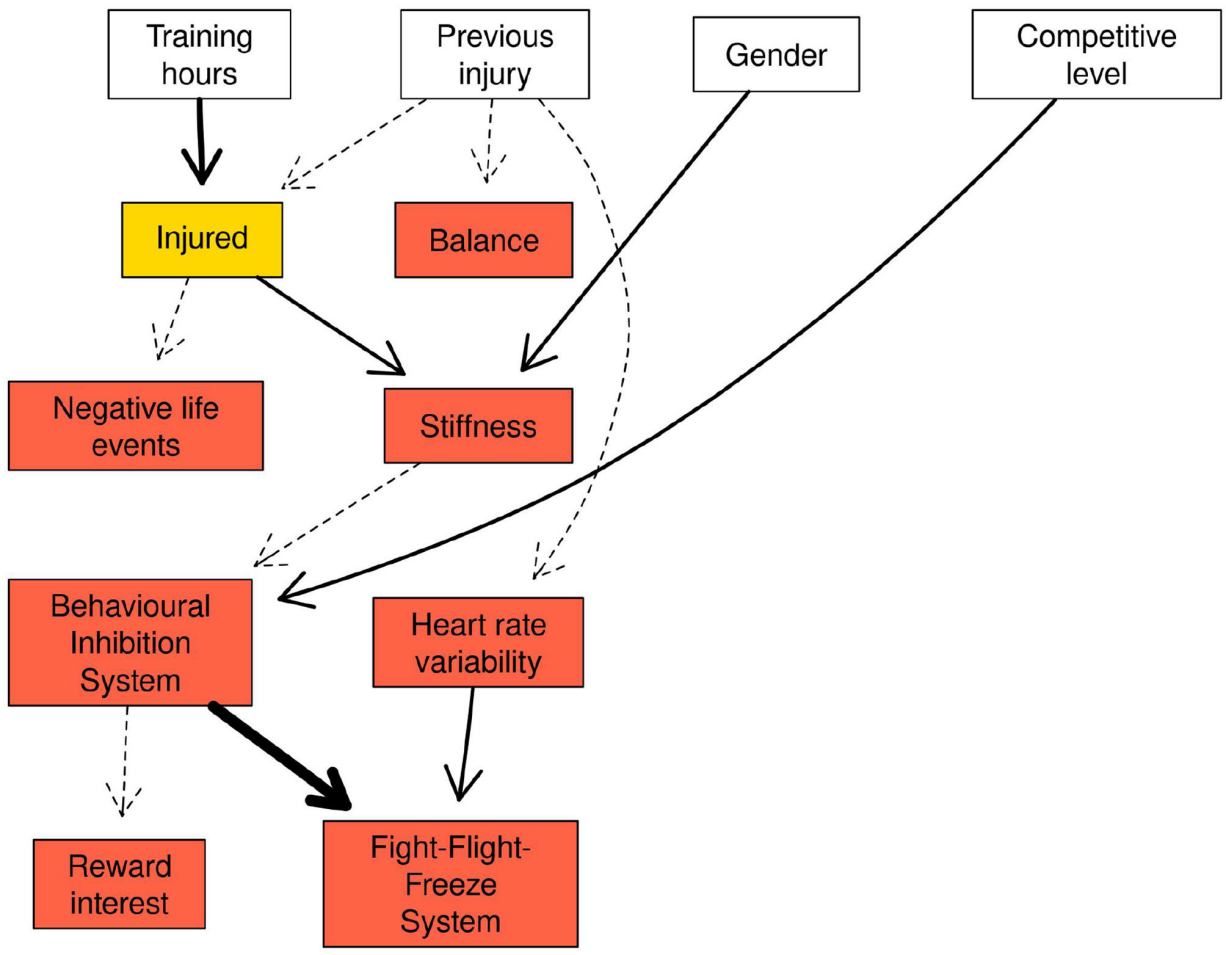
Network structure of the changes within variables between time points.

The second network structure (Figure 4) examined changes within variables between time points and the probability of injury. An arc between Behavioural Inhibition System (BIS) → Fight-Flight-Freeze System (FFFS) with strength 1.00 was present in the network. Arcs between competitive level → BIS and gender → stiffness had a strengths of 0.60 and 0.56 respectively Similarly, the arc between HRV → FFFS was 0.58. The arcs between BIS → FFFS and HRV → FFFS were examined further by drawing random observations from the conditional probability distribution and examining the relationship in a Bayesian linear regression model. A separate linear regression examined the interaction between BIS and HRV.

Results from the Bayesian linear regression model are presented in Table 8 and include 95% credible intervals (CrI). Increases in BIS were associated with increases in FFFS (*b* = −0.19, 95% CrI = [−0.25, −0.13]), whereas positive changes in HRV were associated with decreased changes in FFFS (*b* = 0.41, 95% CrI = [0.36, 0.47]). There was no clear interaction between HRV and BIS (*b* = −0.02, 95% CrI = [−0.08, 0.03]).

**Table 8:** Estimate, error, and 95% credible intervals for the fixed effects in the linear model containing Fight-Flight-Freeze System, Behavioural Inhibition System, and Heart rate variability.

**Term**	**Estimate**	**Error**	**95% CI**
Intercept	0.00	0.03	[−0.05, 0.06]
Behavioural Inhibition System (BIS)	0.41	0.03	[0.36, 0.47]
Heart rate variability (HRV)	−0.19	0.03	[−0.25, −0.13]
BIS:HRV	−0.02	0.03	[−0.08, 0.03]

**Figure 5:**
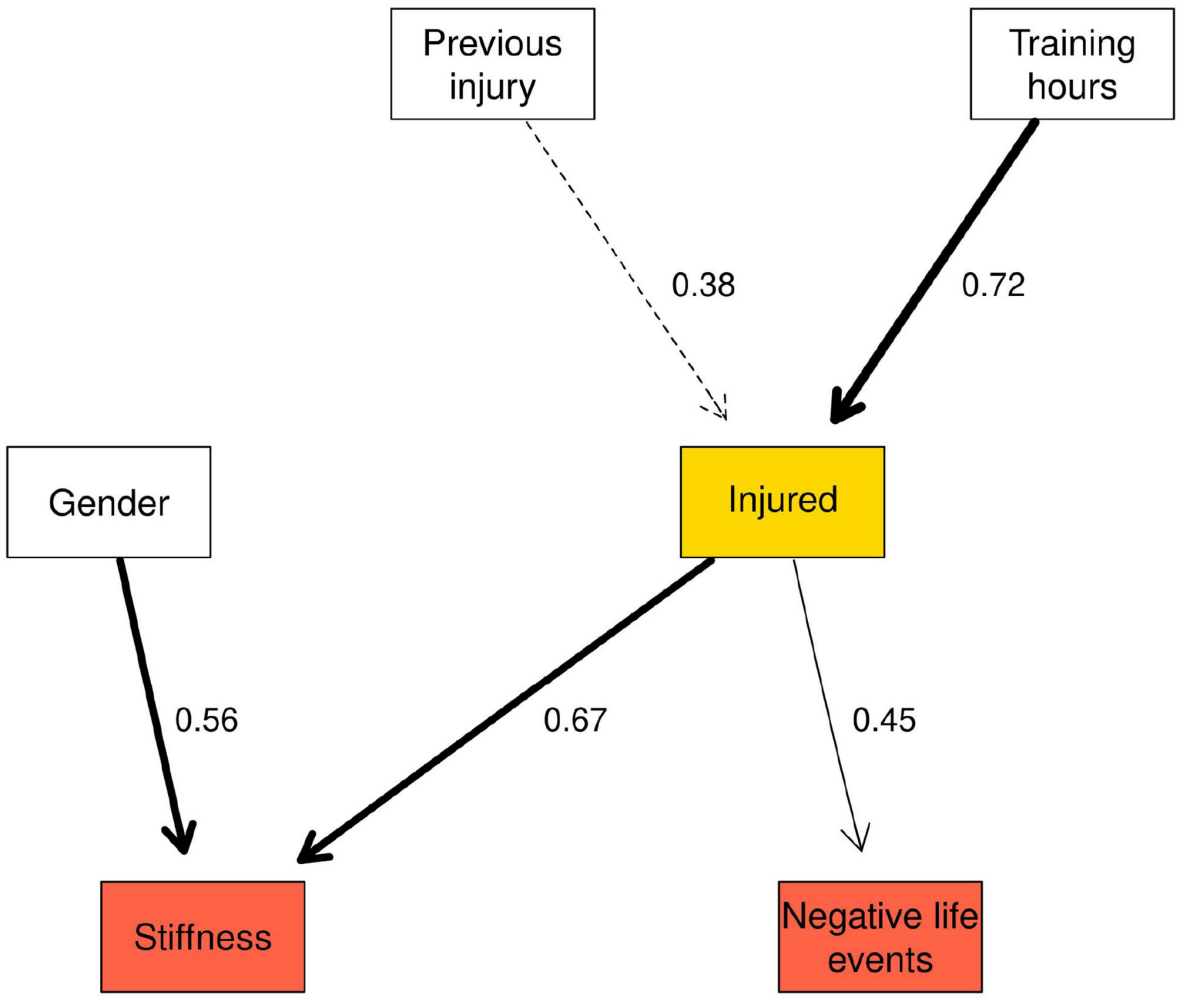
Markov blanket for the injured node in the network reflecting changes within variables between time points.

The Markov blanket for the “injured” node contained previous injury, gender, training hours per week and stiffness and negative life events (NLE; Figure 5). For stiffness and NLE, the values in the nodes represent the standardised change between time points. Combinations of NLE and stiffness at one SD below the mean change, at the mean change, and 1 SD above the mean change are presented in Table 9. Increases in muscle stiffness was found to increase the risk of injury, which was further increased when there were increases in NLE stress. Changes in both NLE and stiffness of 1SD above the mean change resulted in a high probability of being injured (0.71) over the duration of the study. With stiffness held at the mean change, the probability of “injured” rose notably from 0.35 to 0.64 with NLE at 1 SD below an 1 SD above, respectively.

**Table 9:** The probability of injury with values of stiffness and negative life events held at 1 SD below the mean change, at the mean change and 1 SD above the mean change.

**Probability**	**Negative life events**	**Stiffness**
0.71	+1 SD	+1 SD
0.64	+1 SD	Mean
0.62	+1 SD	−1 SD
0.52	Mean	+1 SD
0.44	Mean	Mean
0.43	Mean	−1 SD
0.42	−1 SD	+1 SD
0.35	−1 SD	Mean
0.35	−1 SD	−1 SD

Table 10 shows the three highest and lowest probabilities for injury for all variables in the Markov blanket (full results in Supplementary Table 7). The combination of 1 SD above the mean change for negative life events (NLE) and stiffness and “High” hours per week and previous injury resulted in the highest probability that an injury would be sustained during the study (0.77). In contrast, below average changes in NLE and stiffness combined with “Low” hours per week and no previous injury resulted in the lowest probability of an injury (0.11).

**Table 10:** Highest and lowest probability of injury, conditional on all the variables in the Markov blanket for “injured”.

**Probability**	**Training hours**	**Previous injury**	**Negative life events**	**Stiffness**
**Highest**				
0.77	High	Injury	+1 SD	+1 SD
0.74	High	No injury	+1 SD	+1 SD
0.72	Low	Injury	+1 SD	+1 SD
**Lowest**				
0.15	Low	No injury	−1 SD	+1 SD
0.13	Low	No injury	−1 SD	Mean
0.11	Low	No injury	−1 SD	−1 SD

## Discussion

The study investigated the multifaceted interactions of stress-related variables and injury occurrence using the stress-injury frameworks presented by Williams and Andersen (1998) and Appaneal and Perna (2014), and a prospective, repeated measures design applied to a large cohort of athletes. Relationships between stress-related psychosocial and physiological factors and injury were investigated using two Bayesian network structures; the first was a two-time network that investigated the relationships between variables across time points in the study, and the second network used differential equations to model the changes in variables between time points. The latter facilitated the development of new insights into the interactions of stress-related factors with injury occurrence, exploring changes in both psychosocial and physiological factors that may occur preceding an injury.

The first network revealed several links between the injured nodes and other variables in the network. A combination of high stiffness and poor balance resulted in the highest probability of injury in the Markov blankets for “injured” nodes. The presence of these factors at both injured nodes indicated that the interaction of these variables is important for determining an athlete's risk of injury. In the second network, the highest probability of injury was observed when increases in stiffness and negative life events were both greater than average, indicating that the combination of changes in psychosocial and physiological stress-related factors may combine additively to increase the risk of injury (Appaneal and Perna, 2014).

Of all the variables measured in the study, muscle stiffness appeared to be most strongly related to injury. Both “High” levels of stiffness in the first network, and greater than average increases in stiffness in the second network were found to increase the risk of injury. However, high stiffness may only increase the risk of injury if other factors are also present. To elaborate, the combination of high stiffness *and* poor balance was found to result in the greatest probability of injury. In contrast, athletes with high stiffness and good balance were less likely to be injured, suggesting that improved postural stability may counteract the potential harmful effects of high levels of muscle stiffness. Several studies have identified how balance (Trojian and McKeag, 2006; Romero-Franco et al., 2014) and muscle stiffness (Pickering-Rodriguez et al., 2017; Butler et al., 2003) are separately related to injury. The BN structures examined in this study provided insight into these two stress-related factors and their relation to injury occurrence.

The findings also facilitated an understanding of the interaction between balance and injury. At both injured nodes in the first network, balance was linked to injury. Despite the weak arc strength at both injured nodes, a “High” balance score, which is considered indicative of impaired postural stability (Romero-Franco et al., 2014), was found to increase the probability of injury. This finding is consistent with previous research that has reported an association between decrements in postural stability and increased injury risk (Trojian and McKeag, 2006; Riemann et al., 1999; Romero-Franco et al., 2014). Postural stability is often used as an indicator of athlete performance level, with higher level athletes demonstrating better postural stability over their lower level counterparts (Paillard et al., 2006). Athletes who competed at a higher level were also more likely to have good balance (“Low” balance), compared to their lower level counterparts. These findings suggest that better postural stability is associated with both a higher level of performance and a lower probability of sustaining an injury, reinforcing the importance of postural stability as a feature of athletic training programmes designed to prepare athletes for the demands of high intensity training and competition (Hrysomallis, 2011).

Negative life events captured at a single time point have previously been reported to be most strongly associated with injury (Ivarsson et al., 2017; Williams and Andersen, 2007). The repeated measures approach combined with the second network analysis employed in this study demonstrated that greater than average increases in negative life event stress between time points increased the probability of being injured during the study period. However, negative life event stress had almost no effect on the probability of injury in the first network, which indicated that the relative change in life events may be more important than the absolute score for life events, despite the latter being commonly used in sports injury research to date. For example, an athlete who reports a negative life event score of 1 during the first time point, but then a score of 5 at the second time point will have a 400% increase in their life event score. Although the absolute score would be “Low,” the relative increase between time points may have been caused by a significant event in the athlete's life that had a considerable psychological and physiological effect (Appaneal and Perna, 2014). Future research should therefore consider study designs and analyses that enable relative changes in an individual athlete's life events to be assessed (Ivarsson et al., 2014).

The majority of research has however, consistently identified major life events, particularly those events with a negative valence, as the strongest predictor of injury in Williams and Andersen's model (Ivarsson et al., 2017). During the initial network structure development, no arcs between the negative life event nodes and injured nodes were found by the Tabu search algorithm. Given the reported association between negative life events and injury, an arc was fixed between these variables to allow this relationship to be examined more closely. When negative life events were “High” the probability of injury showed a negligible change at the injured_1 node and decreased by −0.04 at the injured_2 node. One possible explanation for these findings may be due to the use of the LESCA questionnaire in a repeated measures design. In the original LESCA, participants are asked to report major life events that have occurred over the previous 12-months (Petrie, 1992). In this study, athletes completed the LESCA at three time points with an approximate 4-month interval after baseline. Athletes were asked to report any events which had occurred since the previous data collection session in order to avoid inflated scores caused by reporting the same event on multiple occasions. While modifications were made to the LESCA to tailor the items to the study cohort, the use of a shorter 4-month time interval between data collections may have reduced the likelihood for life events listed in the LESCA to have taken place. For example, at the second and third time points, 26% of participants reported 0 negative life events for the preceding 4-month period. Simply, it may be that the items on the LESCA are less suitable for repeated measurements with durations shorter than the advocated 12-months than a measure that captures minor life events (Fawkner et al., 1999).

Williams and Andersen's (1998) model proposed a number of coping resources that were either directly related to injury or moderated the relationship between life stress and injury occurrence; for example, general coping strategies (e.g., good sleeping habits and self-care), social support systems and stress management skills. Although coping was not measured in the current study, several studies have found high levels of social support can reduce the risk of injury (Petrie, 1993; Johnson et al., 2014; Petrie et al., 2014). The lack of association between major/negative life events and injury reported here may be attributed to athletes in this study having the necessary coping resources to mitigate against the effects of any negative life event stress they experienced. Therefore, future research should consider including a measure of coping alongside that of life event stress to help explain the possible moderating effect.

Despite the uncertainty regarding the relationship between injury and heart rate variability (HRV) in the first network, “Low” HRV increased the probability of injury from 0.17 (“High” HRV) to 0.26 (“Low” HRV). This finding is consistent with previous research that has found reduced HRV indices to be indicative of illness or maladaptation to training due to decreased parasymapthic activity, which often precedes injury (Bellenger et al., 2016; Gisselman et al., 2016; Williams et al., 2017). An arc between FFFS_1 and injured_2 (arc strength = 0.40) was also observed in the first network, where the risk of injury was increased from 0.13 to 0.29 with FFFS in the “High” and “Low” states, respectively. Interestingly, the “Low” FFFS score was also related to injuries at subsequent time points. One possible explanation for this finding could be that those athletes who reported “Low” FFFS score were less fearful, and may therefore engage in more risk taking behaviours, increasing the probability of injury. The RST theory proposes that higher levels of FFFS increase avoidance motivation (Corr et al., 2016), and therefore “High” FFFS may have acted as a deterrent from taking risks while training and competing, reducing exposure to situations that could have resulted in injury. The RST theory further proposes that the combination of high BIS and high FFFS is likely to result in a more anxious disposition due to high levels of avoidance and high goal conflict characterised by high levels of FFFS and BIS (Corr, 2013). The first network reported an association between “High” FFFS and “High” BIS, while the second network found an association between increases in FFFS and increases in BIS. High levels of anxiety and anticipation of stressful situations have been linked to reductions in HRV indices including RMSSD (Chalmers et al., 2014; Pulopulos et al., 2018). This association along with the proposed actions of the RST theory (Corr et al., 2016) provides a potential explanation for the negative relationship between FFFS and HRV identified in the second network. To elaborate, high levels of BIS are proposed to be the result of goal conflict, an example of which would be simultaneous triggering of the FFFS (avoidance) and BAS (approach) systems. The goal conflict is likely to elicit a physiological response (e.g., decreased HRV) in preparation to engage in the required behaviour to resolve the goal conflict (Corr et al., 2016). In the present study, however, the role of BAS was limited, as evidenced by the initial network structures in which the BAS had limited connectivity with other components of the network. Consequently, a more detailed examination of the role of RST in the injury process is warranted.

This study had a number of strengths. A major critique of the sport injury literature has been the use of only one wave of measurement that may not be reflective of the dynamic nature of the variables that are associated with injury (Johnson et al., 2014). The longitudinal repeated measures design of the current study allowed changes over time and between time points to be captured and explored. Although there are unique and significant challenges with research employing such designs, a more fine-grained understanding of the dynamic relationships between stress-related factors and injury occurrence in athletes was achieved when compared to traditional cross-sectional, single time point research. As advocated by Johnson et al. (2014), future research should continue to use repeated measure designs, including designs with more frequent monitoring, such as daily and weekly measures to gain further insight into the dynamic interplay between stress-related factors and injury. Sport injury research has also been criticised for adopting analytic approaches that are reductionist in nature (Bittencourt et al., 2016) that fail to account for the complex, emergent behaviour that is characteristic of injury occurrence. The use of an interdisciplinary framework combined with a BN modelling approach in the study facilitated extended insight into the complex interplay that exists between psychosocial and physiological markers of stress and injury occurrence. The BN networks allowed several markers of stress that were free to interact with each other, as well as injury, to be explored.

While the BNs provided a contemporary approach that improved upon traditional methods such as logistic regression (Olmedilla et al., 2018), a number of assumptions were made that potentially limited the approach employed in the study. Firstly, the choice was made to binarise variables in the first network so only “Low” and “High” states were observed. Although binarising variables is a common procedure in BN analysis and has several advantages, Qian and Miltner (2015) highlighted that both a loss of statistical accuracy and potential difficulty in the subsequent interpretation of the model may arise. For example, the meaning of a “Low” and “High” value in this study was only meaningful for the population studied, and there could be additional levels within each category that were not investigated. The use of a median split to determine the “Low” and “High” states could also be improved by using known cut-off scores that are associated with a higher risk of injury. While the measures used in the current research did not have any clear cut-off scores supported by the literature, future research should aim to identify potential cut-off scores that would consequently enable a more meaningful splitting procedure compared to using the group median. Furthermore, in order to collect data on a large sample of participants, suitable measures were required to ensure the viability of the data collection. However, a reduction in the sensitivity of some of these measures may have inhibited our ability to detect more subtle variation in the athletes' responses. For example, a more sensitive measure of postural stability may have been achieved with the use of a force plate, which is considered the gold standard to provide detailed data and enable a more fine-grained analysis (Ross et al., 2011). However, the video-capture approach employed in the study ensured an accessible, non-invasive and readily applied method of capturing the respective measure.

The ability to capture large sample sizes of injured athletes has been recognised as a significant challenge in sports injury research (Ruddy et al., 2019) and affects the success to which stress-injury interactions can be identified and understood. This is further exacerbated in studies that employ longitudinal prospective repeated measures designs where high levels of participant retention are required. Consistent with Williams and Andersen's (1998) stress-injury model, we therefore employed a global definition of injury as the primary outcome measure, to optimise sample size of injury occurrences across the repeated measures prospective study design. As suggested by Ruddy et al. (2019), a larger number of observations and injury events is needed to improve the ability to identify and to make more meaningful predictions. Further conventional injury exposure measures (sport type, training load) were subsequently discretised or reduced for the data capture, which caused some loss of information and has been suggested to limit the ability to capture risk profiles (Carey et al., 2018). Using pooled or discretised definitions for some measures hindered a detailed causal-effect insight into injury-specific risk factors. However, the more holistic, interdisciplinary approach adopted in this study was benefited by the ability to gain and substantiate a more complete picture of the complex, multifaceted stress-injury interactions that exist in sport.

The multifactorial, interdisciplinary approach employed in the study required the selection of stress-related measures derived from a psychosocial and physiological perspective. The variables used in the present study were not definitive. Additional measures relating to coping, injury prevention behaviours, injury-specific biomechanics and stress hormones such as cortisol, which been found to be a marker of both psychological and training-related stress (Perna and McDowell, 1995; Appaneal and Perna, 2014), could help to further elucidate the relationship between stress and injury. Future research should continue to explore the relationship between these measures, as well as their effect on the risk of injury. In particular, there is scope to build upon Appaneal and Perna's (2014) biopsychosocial model of stress, athletic injury and health which provides a framework for examining the interplay between psychological, behavioural, and training-related factors. Future developments in the capture of life event stress using the LESCA are also warranted. Although the LESCA is the most widely used measures of major life events in sports injury research, modifications, including adjustment to the scoring of items are potentially justified to facilitate extended insight into the reported responses. For example, the LESCA may negate vastly different psychological and physiological effects between moderately negative and extremely negative events since there is no way to differentiate between an athlete who has answered four items as moderately negative, and one item as extremely negative. Therefore, future research could develop a modified version of the LESCA that could distinguish between these types of responses and their effects.

Finally, the findings of this study have important practical implications for athletes, coaches, and clinicians in relation to the additive and interactive effects of multiple sources of stress on injury occurrence. Specifically, the study evidenced a combined effect of psychosocial and physiological stress-related factors that could increase the probability of injury to a greater extent than any isolated factor. When assessing an athlete's training plan, readiness to engage in, and recovery from training, coaches and clinicians should employ a risk profile that integrates multifaceted sources of stress. For example, in addition to monitoring training loads and using tools to determine an athlete's physiological status, coaches need to also consider an athlete's psychological state. In particular, when an athlete is facing significant life event stress, adjusted training intensity and volume may be necessitated to support athlete's coping with the additional duress and to subsequently safeguard optimal health and well-being. In essence, injury risk is exacerbated when an athlete is experiencing psychological stress due to exposure to negative life events and exhibiting physiological responses associated with an increased injury potential. The identification of such a “high risk” profile is subsequently important in helping to monitor and reduce injury risk for athletes. For example, while high muscle stiffness is important for optimal performance (Pruyn et al., 2015), this study demonstrated it can heighten injury risk, which is likely to be exacerbated when accompanied by the experience of negative life events by an athlete. In order to understand how an athlete's injury risk may increase over time, it is important to acknowledge the breadth and interaction of stress-related factors that could heighten susceptibility and be receptive to training and life experience changes.

To summarise, this study provided novel insights into the multifaceted nature of the stress-injury relationship using a novel interdisciplinary approach coupled with Bayesian Network analytical techniques. Muscle stiffness and increases in negative life event stress were identified as strong predictors of injury within the multifaceted athlete cohort, while other factors including personality characteristics and postural stability were also found to contribute to the probability of injury occurrence. Future research combining a repeated measures approach and complex analyses of the interactions between multifaceted stress-related measures are advocated to enhance understanding of the injury occurrence in sport.

## Data Availability Statement

The datasets presented in this study can be found in online repositories. The names of the repository/repositories and accession number(s) can be found at: https://github.com/hfshr/frontiers-paper/tree/master/data.

## Ethics Statement

The studies involving human participants were reviewed and approved by Cardiff Metropolitan University. The patients/participants provided their written informed consent to participate in this study.

## Author Contributions

HF, MG, LE, and RM developed the concept for the paper. HF was the lead author for the paper and designed, collected, and analysed that data. MG, LE, and RM were co-authors on the paper. MS provided assistance with data analysis. MB provided assistance with data collection. All authors contributed to the article and approved the submitted version.

## Conflict of Interest

The authors declare that the research was conducted in the absence of any commercial or financial relationships that could be construed as a potential conflict of interest.

## Footnotes

^1^The term “query” in relation to Bayesian Networks stems from Pearl's expert systems theory Pearl (1988). A query can be submitted to an expert (in this case, the network is the expert) to get an opinion, the expert then updates the querier's beliefs accordingly. Widely used texts on Bayesian Network analysis (Koller and Friedman, 2009) have widely adopted the terminology in favor of that used in traditional statistics.
