# Supplementary material for: An Interdisciplinary Examination of Stress and Injury Occurrence in Athletes
Source: Front Sports Act Living. 2020 Dec 14;2:595619. doi: 10.3389/fspor.2020.595619 (PMC7739595; doi:10.3389/fspor.2020.595619)
Supplement: Supplementary file 1 [file Data_Sheet_1.PDF]

S1 Appendix - RST-PQ Validation

The validation of the RST-PQ was examined in a separate programme of PhD research which has yet to be published (1). The present author was involved in the validation process as the participants from this study were used to validate the RST-PQ. Details of validation process are included here for detail but are not strictly part of this programme of research.

To establish the factorial validity of the 65-item model of the RST-PQ in a population of university sports students, Bayesian Structural Equation Modelling (2) was used with responses from sample of 419 university-level athletes (1). BSEM was used for the validation as it several advantages over the maximal likelihood procedures used in traditional confirmatory factor analysis (CFA). In particular, BSEM allows informative variance priors to be specified on cross loadings. In traditional CFA, cross loadings are held at zero and can lead to a blocked or miss-specified model (3). By recognising that some small cross-loadings and correlated residuals could be present within the items, BSEM can reduce the possibility of model misspecification (4). To perform the validation, three models were specified following the recommendations of (2). Model one specified non-informative priors on the factor loadings and exact zeros on the cross-loadings and correlated residuals. Model two specified non-informative priors on the factor loadings, informative approximate zeros on the cross-loadings and exact zeros on the correlated residuals.

Lastly, model three specified non-informative priors on the factor loadings, and informative approximate zeros on the cross-loadings and correlated residuals. The variance of the informative priors was set at $\pm$ 0.10, which equates to loadings with 95% limit of $\pm$ .20 and implies weak cross loading and correlated residuals. Loadings that were outside of the range were identified as being highly correlated or having a large source of residual variance (2).

Estimation of the BSEM models was performed in MPLUS where a Markov chain Monte Carlo (MCMC)

algorithm was used with a Gibbs sampler, in which 100,000 iterations were drawn to examine the parameter estimates and model convergence. Model convergence was assessed using potential scale reduction factor (PSRF), where values between 1.0 and 1.1 indicated good model convergence ((5); (6)). Model fit was assessed by Posterior Predictive P (PPP) values and 95 % credibility intervals, where PPP values >= 0.50 and 95 % confidence interval balanced around approximately zero indicated good model fit (2) To compare the different BSEM models, the deviance information criterion (DIC) was used, where lower values indicate a better model-fit.

The analysis revealed the 65 item RST-PQ to have both good model convergence and model fit. In addition, all the main factor loadings were significant. However, several problematic items were identified based on weak factor loadings and substantial cross-loadings and correlated residuals. Consequently, the model was re-specified following an item deletion process (1) and a 51-item RST-PQ emerged with acceptable model-fit (PPP = .739, 95% posterior predictive confidence intervals = -199.220, 101.356). The findings from the BSEM were replicated with a sample of 350 participants from the current research programme (PPP = .787, 95% posterior predictive confidence intervals = -208.405, 90.785). Consequently, the 51-item questionnaire was adopted as the chosen measure of RST in the current research.

References

1. Young T. Validation of the revised Reinforcement Sensitivity Theory - Personality Questionnaire (RST-PQ).

2. Muthén B, Asparouhov T. Bayesian structural equation modelling: A more flexible representation of substantive theory. *Psychological Methods* (2012) **17**:313–335. doi:10.1037/a0026802

3. Marsh HW, Muthén B, Asparouhov T, Lüdtke O, Robitzsch A, Morin AJS, et al. Exploratory structural equation modeling, integrating CFA and EFA: Application to students' evaluations of university teaching. *Structural Equation Modeling* (2009) **16**:439–476. doi:10.1080/10705510903008220

4. Gucciardi D, Zyphur M. "Exploratory structural equation modelling and Bayesian estimation," in *An introduction to intermediate and advanced analyses for sport and exercise scientists*, eds. N. Ntoumanis, N. Myers (London: Wiley), 172–194.

5. Gelman A, Brooks SP. General methods for monitoring convergence of iterative simulations. *Journal of Computational and Graphical Statistics* (1998) **7**:434–455.

6. Gelman A, Carlin JB, Stern HS, Dunson DB, Vehtari A, Rubin DB. *Bayesian Data Analysis.* 3rd ed. Boca Raton: Chapman; Hall/CRC. (2013).
