# Supplementary material for: An Interdisciplinary Examination of Stress and Injury Occurrence in Athletes
Source: Front Sports Act Living. 2020 Dec 14;2:595619. doi: 10.3389/fspor.2020.595619 (PMC7739595; doi:10.3389/fspor.2020.595619)
Supplement: Supplementary file 2 [file Data_Sheet_2.PDF]

S1 Table. Summary of modifications to LESCA items.

Q	Original	Students	Non-students
19	Beginning a new school experience (beginning college, transferring college etc)	Beginning a new school experience (beginning university, transferring university etc)	Beginning a new work experience
21	Academic probation / ineligibility	*No change*	Removed
22	Being dismissed from dorm or other residence	Being dismissed from halls or other residence	Being asked to vacate house/home
27	Financial problems concerning school	*No change*	Financial problems
29	Conflict with room-mate	*No change*	Conflict within household
36	Suspended from team for non-academic reasons	*No change*	Removed
49	Being absent from school (classes) because of participation in sport	Being absent from university (classes) because of participation in sport	Being absent from work because of participation in sport
61	Major change in level of academic performance (doing better or worse)	*No change*	Major change in level of performance at work (doing better or worse)
62	Making career decisions (applying to graduate school,interviewing for jobs, etc)	Making career decisions (applying for Masters degree, interviewing for jobs, etc)	Making career decisions (applying for Masters degree, interviewing for jobs, etc)
