# Supplementary material for: An Interdisciplinary Examination of Stress and Injury Occurrence in Athletes
Source: Front Sports Act Living. 2020 Dec 14;2:595619. doi: 10.3389/fspor.2020.595619 (PMC7739595; doi:10.3389/fspor.2020.595619)
Supplement: Supplementary file 3 [file Data_Sheet_3.PDF]

S2 Table. All arc strengths greater than 0.3 included in the first network.

from	to	strength	direction
Baseline NLE	Negative life events_1	1.00	1.00
Baseline NLE	Negative life events_2	1.00	1.00
Negative life events_1	Injured_1	1.00	1.00
Negative life events_2	Injured_2	1.00	1.00
Behavioural Inhibition System_1	Fight-Flight- Freeze System_1	0.98	0.70
Fight-Flight- Freeze System_1	Behavioural Inhibition System_1	0.98	0.30
Sport type	Training hours	0.94	0.50
Training hours	Sport type	0.94	0.50
Baseline NLE	Reward Interest_1	0.84	1.00
Sport type	Baseline NLE	0.78	1.00
Behavioural Inhibition System_2	Fight-Flight- Freeze System_2	0.74	0.71
Fight-Flight- Freeze System_2	Behavioural Inhibition System_2	0.74	0.29
Gender	Stiffness_1	0.71	1.00
Previous injury	Stiffness_1	0.57	1.00
Negative life events_1	Behavioural Inhibition System_1	0.55	0.97
Behavioural Inhibition System_1	Negative life events_1	0.55	0.03
Fight-Flight- Freeze System_1	Behavioural Inhibition System_2	0.52	1.00
Competitive level	Balance_1	0.47	1.00
Injured_2	Stiffness_2	0.46	0.66
Stiffness_2	Injured_2	0.46	0.34
Injured_1	Stiffness_1	0.46	0.36
Stiffness_1	Injured_1	0.46	0.64
Gender	Stiffness_2	0.43	1.00
Fight-Flight- Freeze System_1	Injured_2	0.42	1.00
Training hours	Injured_1	0.42	1.00
Competitive level	Reward Interest_2	0.42	1.00
Baseline NLE	Heart rate variability_1	0.41	1.00
Training hours	Heart rate variability_1	0.40	1.00
Heart rate variability_1	Negative life events_2	0.39	1.00
Gender	Fight-Flight- Freeze System_1	0.37	1.00
Injured_2	Heart rate variability_2	0.37	0.89
Negative life events_2	Behavioural Inhibition System_2	0.37	0.96
Behavioural Inhibition System_2	Negative life events_2	0.37	0.04
Heart rate variability_2	Injured_2	0.37	0.11
Previous injury	Negative life events_2	0.36	1.00
Reward Interest_1	Behavioural Inhibition System_1	0.36	0.41
Behavioural Inhibition System_1	Reward Interest_1	0.36	0.59
Baseline NLE	Reward Interest_2	0.36	1.00
Injured_1	Balance_1	0.36	0.70
Balance_1	Injured_1	0.36	0.30
Injured_2	Balance_2	0.34	0.94
Balance_2	Injured_2	0.34	0.06
Reward Interest_1	Balance_1	0.33	0.22
Balance_1	Reward Interest_1	0.33	0.78
Gender	Fight-Flight- Freeze System_2	0.33	1.00
Previous injury	Competitive level	0.32	0.50
Competitive level	Previous injury	0.32	0.50
Sport type	Fight-Flight- Freeze System_1	0.31	1.00

S3 Table continued

from	to	strength	direction
Behavioural Inhibition System_1	Fight-Flight- Freeze System_2	0.31	1.00
Previous injury	Sport type	0.30	0.46
Sport type	Previous injury	0.30	0.54
