# Supplementary material for: An Interdisciplinary Examination of Stress and Injury Occurrence in Athletes
Source: Front Sports Act Living. 2020 Dec 14;2:595619. doi: 10.3389/fspor.2020.595619 (PMC7739595; doi:10.3389/fspor.2020.595619)
Supplement: Supplementary file 4 [file Data_Sheet_4.PDF]

S3 Table. All arc strengths greater than 0.3 included in the second network.

from	to	strength	direction
Behavioural Inhibition System	Fight-Flight- Freeze System	1.00	0.76
Fight-Flight- Freeze System	Behavioural Inhibition System	1.00	0.25
Training hours	Injured	0.72	1.00
Injured	Stiffness	0.67	1.00
Competitive level	Behavioural Inhibition System	0.60	1.00
Heart rate variability	Fight-Flight- Freeze System	0.58	0.69
Fight-Flight- Freeze System	Heart rate variability	0.58	0.31
Gender	Stiffness	0.56	1.00
Injured	Negative life events	0.45	1.00
Previous injury	Balance	0.43	1.00
Previous injury	Injured	0.38	1.00
Stiffness	Behavioural Inhibition System	0.38	0.93
Behavioural Inhibition System	Stiffness	0.38	0.07
Reward interest	Behavioural Inhibition System	0.35	0.29
Behavioural Inhibition System	Reward interest	0.35	0.71
Previous injury	Heart rate variability	0.33	1.00
