# Supplementary material for: An Interdisciplinary Examination of Stress and Injury Occurrence in Athletes
Source: Front Sports Act Living. 2020 Dec 14;2:595619. doi: 10.3389/fspor.2020.595619 (PMC7739595; doi:10.3389/fspor.2020.595619)
Supplement: Supplementary file 5 [file Data_Sheet_5.PDF]

S4 Table. Breakdown of injury count by sport and injury type (chronic or acute)

sport	acute	chronic
athletics	43.9% (18)	56.1% (23)
basketball	75.0% (6)	25.0% (2)
cricket	40.0% (4)	60.0% (6)
football	76.9% (20)	23.1% (6)
gym	25.0% (1)	75.0% (3)
hockey	88.9% (16)	11.1% (2)
netball	72.7% (8)	27.3% (3)
other	50.0% (3)	50.0% (3)
rugby	88.7% (47)	11.3% (6)
