# Supplementary material for: An Interdisciplinary Examination of Stress and Injury Occurrence in Athletes
Source: Front Sports Act Living. 2020 Dec 14;2:595619. doi: 10.3389/fspor.2020.595619 (PMC7739595; doi:10.3389/fspor.2020.595619)
Supplement: Supplementary file 6 [file Data_Sheet_6.PDF]

**S5 Table. Breakdown of injury count by sport and injury location**

| sport | foot / ankle | lower leg | knee | upper leg | hip | torso | upper arm / shoulder | elbow / lower arm | head / neck |
|---|---|---|---|---|---|---|---|---|---|
| athletics | 36.6% (15) | 14.6% (6) | 9.8% (4) | 17.1% (7) | 7.3% (3) | 9.8% (4) | 4.9% (2) | 0.0% (0) | 0.0% (0) |
| basketball | 25.0% (2) | 0.0% (0) | 12.5% (1) | 0.0% (0) | 0.0% (0) | 37.5% (3) | 25.0% (2) | 0.0% (0) | 0.0% (0) |
| cricket | 0.0% (0) | 10.0% (1) | 20.0% (2) | 30.0% (3) | 0.0% (0) | 30.0% (3) | 0.0% (0) | 10.0% (1) | 0.0% (0) |
| football | 15.4% (4) | 7.7% (2) | 15.4% (4) | 26.9% (7) | 0.0% (0) | 0.0% (0) | 15.4% (4) | 3.8% (1) | 15.4% (4) |
| gym | 0.0% (0) | 0.0% (0) | 25.0% (1) | 0.0% (0) | 25.0% (1) | 25.0% (1) | 25.0% (1) | 0.0% (0) | 0.0% (0) |
| hockey | 27.8% (5) | 16.7% (3) | 11.1% (2) | 22.2% (4) | 0.0% (0) | 5.6% (1) | 0.0% (0) | 11.1% (2) | 5.6% (1) |
| netball | 36.4% (4) | 0.0% (0) | 27.3% (3) | 9.1% (1) | 9.1% (1) | 9.1% (1) | 9.1% (1) | 0.0% (0) | 0.0% (0) |
| other | 16.7% (1) | 0.0% (0) | 16.7% (1) | 0.0% (0) | 0.0% (0) | 50.0% (3) | 16.7% (1) | 0.0% (0) | 0.0% (0) |
| rugby | 18.9% (10) | 3.8% (2) | 26.4% (14) | 17.0% (9) | 5.7% (3) | 1.9% (1) | 17.0% (9) | 0.0% (0) | 9.4% (5) |
| Total | 23.2% (41) | 7.9% (14) | 18.1% (32) | 17.5% (31) | 4.5% (8) | 9.6% (17) | 11.3% (20) | 2.3% (4) | 5.6% (10) |
