# Supplementary material for: An Interdisciplinary Examination of Stress and Injury Occurrence in Athletes
Source: Front Sports Act Living. 2020 Dec 14;2:595619. doi: 10.3389/fspor.2020.595619 (PMC7739595; doi:10.3389/fspor.2020.595619)
Supplement: Supplementary file 7 [file Data_Sheet_7.PDF]

S6 Table. Probabilities of injury conditional on the variables in the Markov blanket for injured_1.

Probability	Competitive level	Training hours	Negative life events_1	stiffness_1	balance_1
0.53	club_university_county	High	Low	High	High
0.46	national_international	High	Low	High	Low
0.44	national_international	High	Low	High	High
0.39	club_university_county	Low	High	High	High
0.37	club_university_county	High	Low	High	Low
0.32	national_international	Low	High	High	Low
0.32	club_university_county	High	High	High	High
0.31	club_university_county	High	High	Low	High
0.3	national_international	Low	High	High	High
0.29	club_university_county	Low	High	Low	High
0.25	national_international	High	High	High	Low
0.25	national_international	High	High	Low	Low
0.25	club_university_county	Low	High	High	Low
0.24	national_international	High	High	Low	High
0.24	national_international	High	High	High	High
0.24	national_international	Low	High	Low	Low
0.22	national_international	Low	High	Low	High
0.19	club_university_county	High	High	Low	Low
0.19	club_university_county	High	High	High	Low
0.18	club_university_county	Low	High	Low	Low
0.17	club_university_county	Low	Low	High	High
0.16	club_university_county	High	Low	Low	High
0.13	national_international	Low	Low	High	Low
0.12	national_international	High	Low	Low	Low
0.12	national_international	Low	Low	High	High
0.12	national_international	High	Low	Low	High
0.09	club_university_county	Low	Low	High	Low
0.09	club_university_county	High	Low	Low	Low
0.07	club_university_county	Low	Low	Low	High
0.06	national_international	Low	Low	Low	Low
0.05	national_international	Low	Low	Low	High
0.04	club_university_county	Low	Low	Low	Low
