# Supplementary material for: An Interdisciplinary Examination of Stress and Injury Occurrence in Athletes
Source: Front Sports Act Living. 2020 Dec 14;2:595619. doi: 10.3389/fspor.2020.595619 (PMC7739595; doi:10.3389/fspor.2020.595619)
Supplement: Supplementary file 8 [file Data_Sheet_8.PDF]

S7 Table. Probabilities of injury conditional on the variables in the Markov blanket for injured_2.

Probability	Fight-Flight-Freeze System_1	Negative life events_2	Stiffness_2	Heart rate variability_2	Balance_2
0.53	Low	Low	High	Low	High
0.46	Low	High	High	Low	High
0.41	Low	Low	High	High	High
0.39	Low	Low	High	Low	Low
0.35	Low	High	High	High	High
0.33	Low	High	High	Low	Low
0.32	Low	Low	Low	Low	High
0.28	Low	Low	High	High	Low
0.26	Low	High	Low	Low	High
0.25	High	Low	High	Low	High
0.23	Low	High	High	High	Low
0.23	Low	Low	Low	High	High
0.22	High	High	High	Low	High
0.21	Low	Low	Low	Low	Low
0.18	Low	High	Low	High	High
0.17	High	Low	High	High	High
0.17	Low	High	Low	Low	Low
0.16	High	Low	High	Low	Low
0.15	High	High	High	High	High
0.14	Low	Low	Low	High	Low
0.13	High	High	High	Low	Low
0.13	High	Low	Low	Low	High
0.11	Low	High	Low	High	Low
0.11	High	High	Low	Low	High
0.11	High	Low	High	High	Low
0.09	High	High	High	High	Low
0.08	High	Low	Low	High	High
0.08	High	Low	Low	Low	Low
0.07	High	High	Low	High	High
0.06	High	High	Low	Low	Low
0.05	High	Low	Low	High	Low
0.04	High	High	Low	High	Low
