# Supplementary material for: An Interdisciplinary Examination of Stress and Injury Occurrence in Athletes
Source: Front Sports Act Living. 2020 Dec 14;2:595619. doi: 10.3389/fspor.2020.595619 (PMC7739595; doi:10.3389/fspor.2020.595619)
Supplement: Supplementary file 9 [file Data_Sheet_9.PDF]

S8 Table. Highest and lowest probability of injury, conditional on the all variables in the Markov blanket for "injured".

Probability	Training hours	Previous injury	Negative life events	Stiffness
0.77	High	injury	+1SD	+1SD
0.74	High	no injury	+1SD	+1SD
0.72	Low	injury	+1SD	+1SD
0.70	High	injury	+1SD	mean
0.69	High	injury	+1SD	-1SD
0.68	High	no injury	+1SD	mean
0.66	High	no injury	+1SD	-1SD
0.65	Low	injury	+1SD	mean
0.64	Low	injury	+1SD	-1SD
0.61	High	injury	mean	+1SD
0.58	High	no injury	mean	+1SD
0.54	Low	injury	mean	+1SD
0.54	High	injury	mean	mean
0.51	High	injury	-1SD	+1SD
0.51	High	injury	mean	-1SD
0.50	High	no injury	mean	mean
0.48	High	no injury	mean	-1SD
0.48	Low	injury	mean	mean
0.47	High	no injury	-1SD	+1SD
0.46	Low	injury	mean	-1SD
0.44	Low	injury	-1SD	+1SD
0.44	High	injury	-1SD	mean
0.42	High	injury	-1SD	-1SD
0.41	High	no injury	-1SD	mean
0.38	High	no injury	-1SD	-1SD
0.37	Low	no injury	+1SD	+1SD
0.37	Low	injury	-1SD	mean
0.35	Low	injury	-1SD	-1SD
0.32	Low	no injury	+1SD	mean
0.29	Low	no injury	+1SD	-1SD
0.20	Low	no injury	mean	+1SD
0.18	Low	no injury	mean	mean
0.16	Low	no injury	mean	-1SD
0.15	Low	no injury	-1SD	+1SD
0.13	Low	no injury	-1SD	mean
0.11	Low	no injury	-1SD	-1SD
