# Supplementary figures and images for: An Interdisciplinary Examination of Stress and Injury Occurrence in Athletes
Source: Front Sports Act Living. 2020 Dec 14;2:595619. doi: 10.3389/fspor.2020.595619 (PMC7739595; doi:10.3389/fspor.2020.595619)

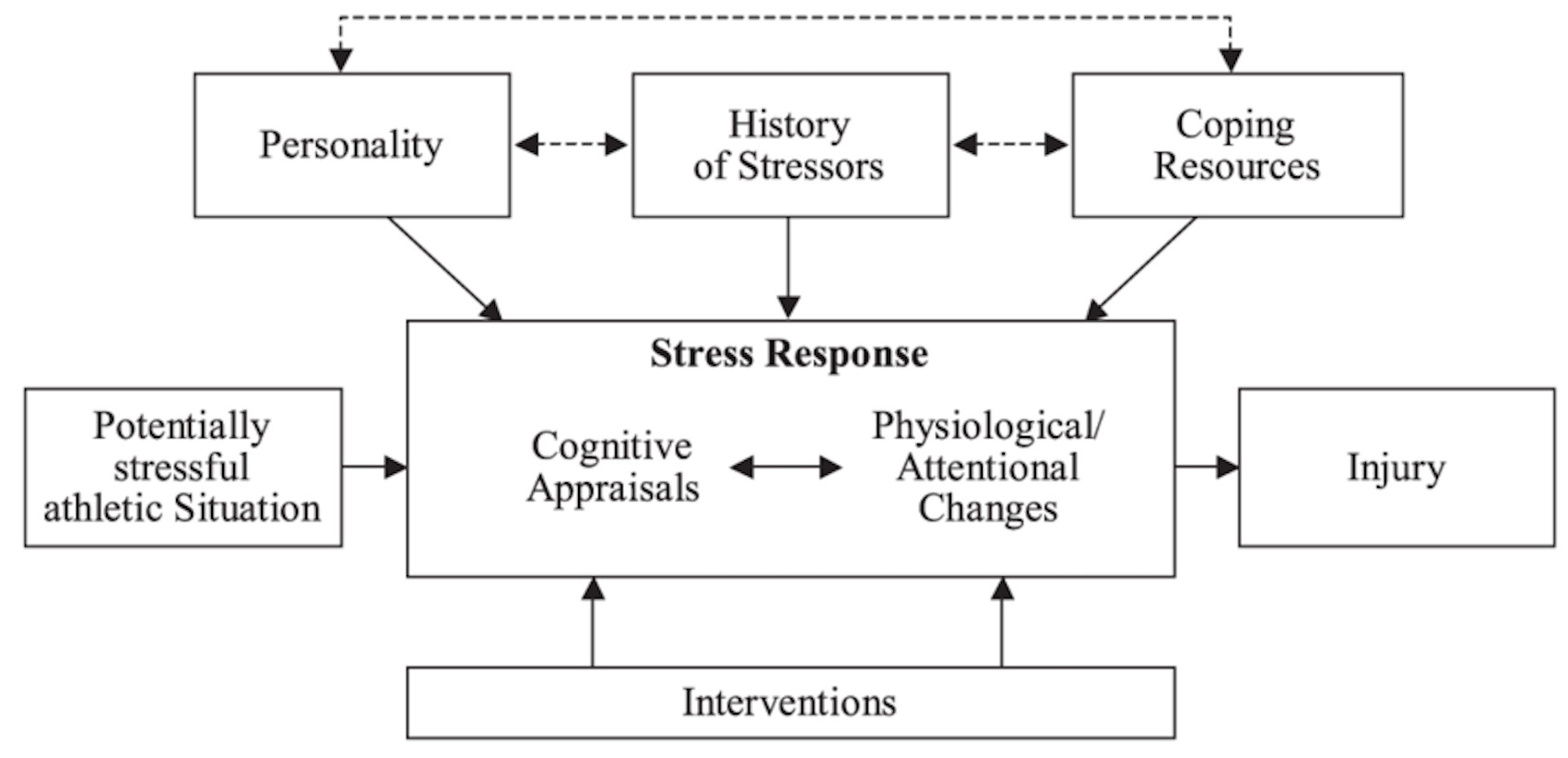

Supplement: Supplementary file 10 [file Image_1.JPEG]

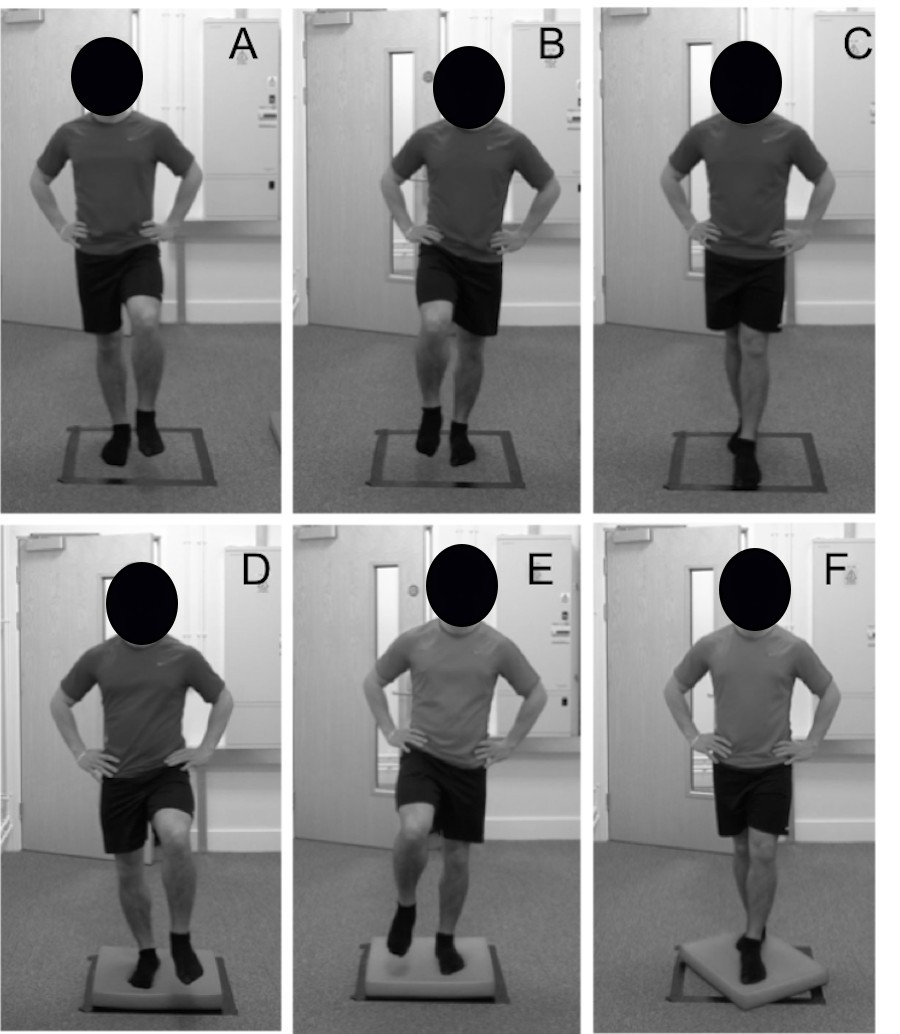

Supplement: Supplementary file 11 [file Image_2.JPEG]

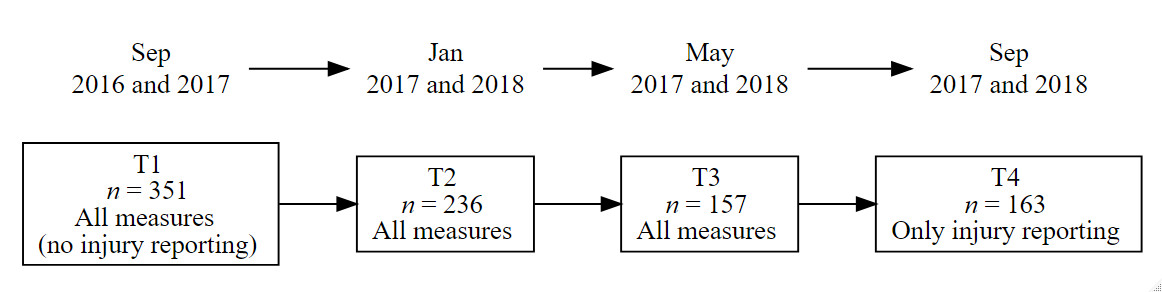

Supplement: Supplementary file 12 [file Image_3.JPEG]

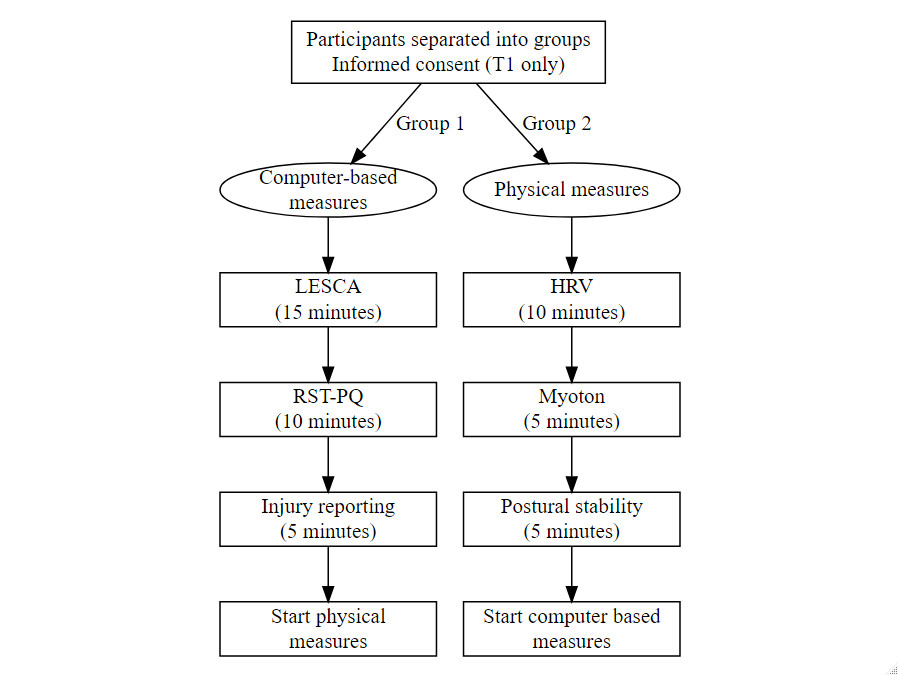

Supplement: Supplementary file 13 [file Image_4.JPEG]
